# Further Studies on the Pyrolytic Domino Cyclization of Stabilized Phosphonium Ylides Bearing an *Ortho*-Aminophenyl Group

**DOI:** 10.3390/molecules23092153

**Published:** 2018-08-27

**Authors:** R. Alan Aitken, Lorna Murray, Alexandra M. Z. Slawin

**Affiliations:** EaStCHEM School of Chemistry, University of St Andrews, North Haugh, St Andrews, Fife KY16 9ST, UK; Lorna.Murray@ed.ac.uk (L.M.); amzs@st-and.ac.uk (A.M.Z.S.)

**Keywords:** phosphonium ylide, pyrolysis, quinoline, phenanthridine, benzocarbazole, X-ray structure

## Abstract

Four new, stabilized phosphonium ylides containing a 2-(benzyl(methyl)amino)phenyl group have been prepared and characterized and are found, upon pyrolysis under gas-phase flow conditions, to lose Ph_3_PO and benzyl radicals to afford new heterocyclic products resulting from domino cyclization of both *C*- and *N*-centered radicals. Most products arise from processes of the former type and have quinoline, phenanthridine, or ring-fused phenanthridine structures, while in one case, a process of the latter type leads to a benzocarbazole product. The X-ray structure of a 2-(methyl(tosyl)amino)phenyl ylide is also reported.

## 1. Introduction

**Scheme 1:**
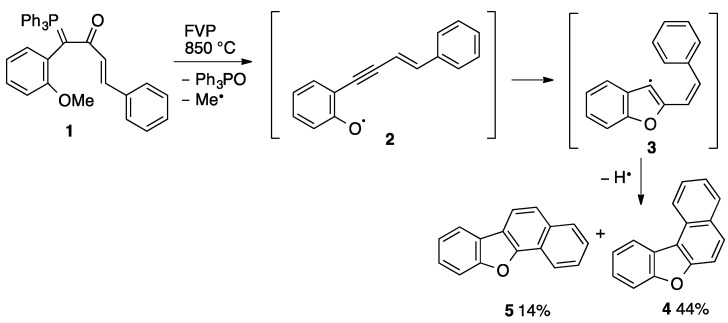
Example of the domino cyclization approach to fused-ring heterocycles.

The synthetic use of flash vacuum pyrolysis (FVP) is now well-established and provides the method of choice to obtain certain, otherwise inaccessible products [1]. Particularly in the area of heterocyclic chemistry, the method has been applied to a wide variety of reaction types and product classes [2]. The inherent advantages of a clean, solvent-, reagent-, and catalyst-free method are further enhanced if several reaction steps can be achieved in sequence in a single gas-phase flow pyrolysis process, which is a good example of the “domino” reaction approach [3,4,5]. In previous studies we have developed a series of methods for the synthesis of fused-ring heterocyclic compounds by FVP of suitably designed carbonyl-stabilized phosphonium ylides. The principle is demonstrated by the case of ylide **1**, which, at 850 °C, eliminates both triphenylphosphine oxide and a methyl radical (Scheme 1). The resulting alkynylphenoxyl radical **2** undergoes spontaneous 5-*endo-dig* cyclization to give the benzofuryl radical **3**, bearing a suitably-placed styryl group to undergo (after *E*/*Z* isomerization) an intramolecular S_H_Ar process, leading to the fused ring product naphtho[2,1-*b*]benzofuran **4** in 44% isolated yield [6,7]. Interestingly, in this case a second isomeric product **5** could also be identified in 14% yield resulting from a rearrangement of **3** prior to cyclization.

**Scheme 2:**
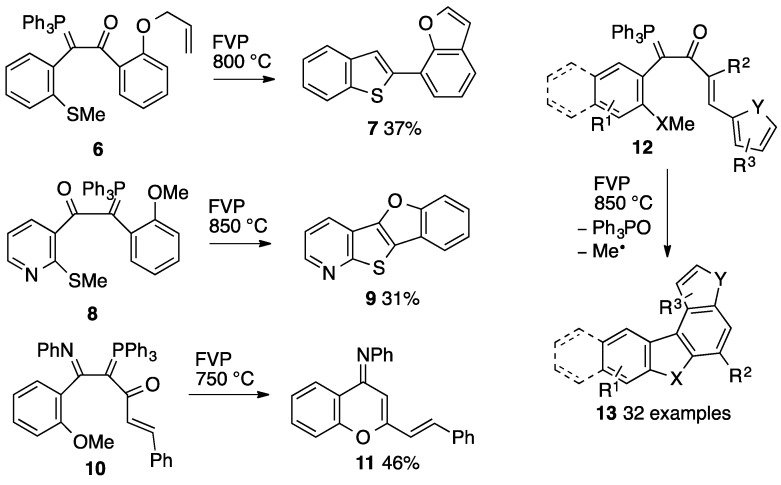
The range of previously reported cyclizations involving O or S.

The method was later extended to a wide range of examples, including an eight-stage cascade process leading from allyloxy ylide **6** to the 7-(2-benzothienyl)benzofuran **7** (Scheme 2) [8], formation of thieno[2,3-*b*]pyridine products including the benzofurothienopyridine **9** from ylide **8** [9], formation of a iminobenzopyranone **11** from ylide **10** [10], and synthesis of a wide range of products with structure **13,** including 24 different fused-ring systems from the general starting ylide structure **12** [11].

**Scheme 3:**
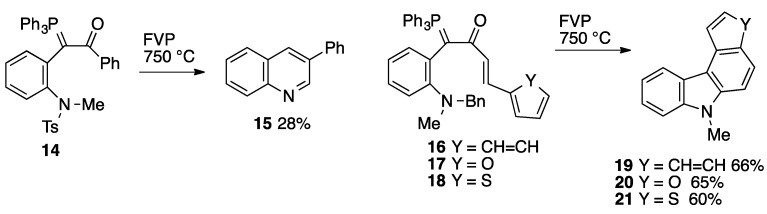
Divergent outcomes resulting from cyclization involving C or N.

All the preceding examples rely on cyclization of phenoxyl or thiophenoxyl radicals, and extension of the method to ylides with a nitrogen-based cyclizing radical introduces an unexpected complication. Preparation of the ylides requires a carbon-based protecting group on nitrogen as well as the leaving group, and transfer of the reactive site from N to C may lead to different products. Thus, while simpler ylides, such as the *N*-methyl-*N*-tosyl compound **14,** give the 3-substituted quinoline **15** resulting from cyclization of –NH-CH_2_ (Scheme 3), the more extended cinnamoyl-type analogues **16**–**18** do undergo the expected cyclization of -N(Me) to give ring-fused carbazole products, **19**–**21** [12].

**Scheme 4:**
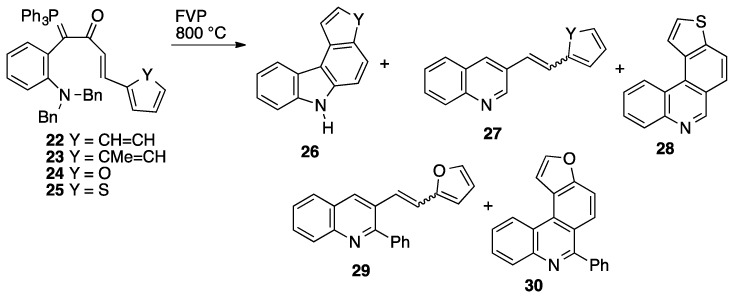
Heterocyclic products derived from *N*,*N*-dibenzyl ylides.

However, when *N*,*N*-dibenzyl ylides **22**–**25** were examined, a more complex picture emerged [13] with all four examples giving mainly the styrylquinoline products **27** (Scheme 4) as mixtures of (*Z*)- and (mainly) (*E*)-isomers, together with the ring-fused carbazoles **26** for ylides **22** and **25** only, and the product **28** resulting from further cyclization of **27** from **25** only. The loss of a phenyl group in formation of products **27** and **28** is notable, and only for compound **24** were the products **29** and **30** retaining the *N*-benzyl-derived phenyl obtained.

It is clear from these results that it is not yet possible to predict the pyrolysis outcome for the amino-functionalized ylides with any degree of certainty and further work is required to fully understand the processes involved. In this paper, we describe the synthesis and characterization of four new amino-functionalized ylides and the results of their pyrolysis, which leads to the formation of seven new fused-ring heterocyclic products.

## 2. Results

### 2.1. X-ray Structure Determination of Ylide ***14***

**Figure 1:**
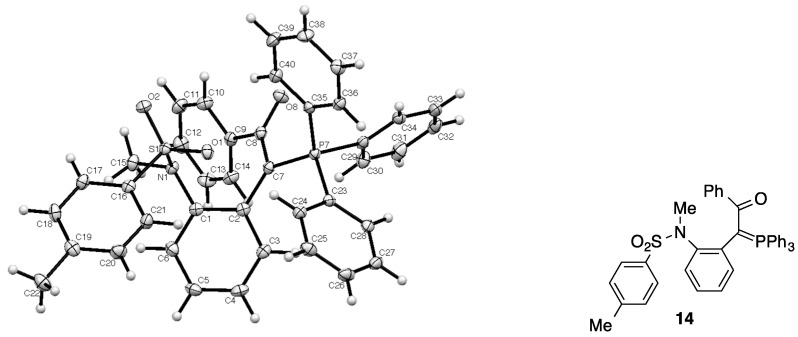
X-ray structure of ylide **14** (ORTEP diagram, 50% level).

Since many of the previously studied nitrogen-containing ylides were formed in disappointing yield and showed severe restricted rotation leading to very broad signals in their room temperature NMR spectra [12,13], it was clear that steric hindrance may be an important factor in their reactivity. To examine this further, we have determined the structure of the *N*-methyl-*N*-tosyl ylide **14** [12] by X-ray diffraction. The resulting structure (Figure 1) confirms that this is indeed a sterically congested molecule with the two very bulky ortho substituents on the central benzene ring scarcely able to fit. Within the crystal there are no significant intermolecular interactions. The following bond lengths within the keto ylide functionality: P(7)-C(7) 1.746(2), C(7)-C(8) 1.408(2), C(8)-O(8) 1.263(2) A are indicative of a good degree of delocalization and a high contribution from the charge-separated structure with P^+^-C=C-O^−^ rather than P=C-C=O. The torsion angle P(7)-C(7)-C(8)-O(8) is −9.0(2), which is within the range normally expected for keto-stabilized ylides [14,15], and indicates a high probability of successful thermal extrusion of Ph_3_PO, as does the low value of 6 Hz [12] for ^2^*J*_P–CO_.

### 2.2. Preparation and Characterization of Ylides ***35**–**38***

**Scheme 5:**
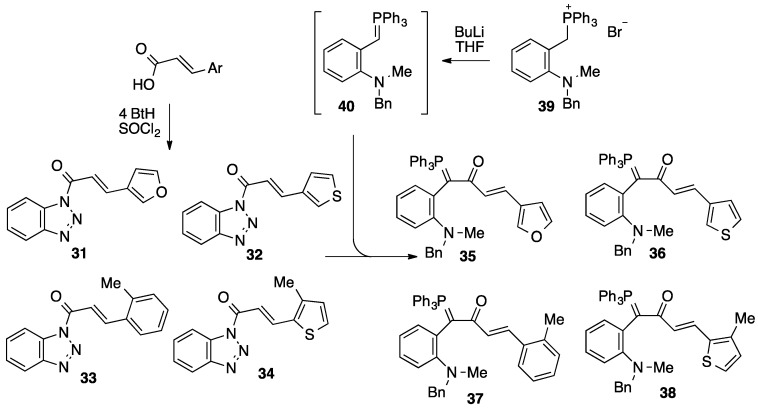
Preparation of ylides **35**–**38**.

The four new ylides chosen for study have structures **35**–**38** (Scheme 5). Compounds **35** and **36** are obviously isomeric with ylides **17** and **18**, which were well-behaved upon pyrolysis and gave the ring-fused carbazoles [12], but whose dibenzyl analogues **24** and **25** showed more varied and unpredictable behaviour [13]. The 2-methylcinnamoyl ylide **37** is similarly the analogue of dibenzyl ylide **23**, and both it and its thiophene analogue **38** are of additional interest, since we previously observed cyclization involving the methyl group to give vinylnaphthalenes and further cyclized products when ylides analogous to **37** and **38** but only lacking the benzylmethylamino group were subjected to FVP [16,17]. As we described previously, it was advantageous to prepare the ylides using *N*-acylbenzotriazoles as the acylating agents [18] rather than acid chlorides, and the four required benzotriazole derivatives **31**–**34** (see Supplementary Material S1–S7) were prepared by treatment of the appropriate carboxylic acids with thionyl chloride (1 equiv.) and benzotriazole (4 equiv.).

The four ylides were prepared as shown in Scheme 5 by treatment of phosphonium salt **39** [12] with butyllithium to generate the ylide **40**, followed by addition of the appropriate *N*-acylbenzotriazole, and were obtained as stable crystalline solids with high melting points. The signals due to the *P*-phenyl groups in the ^1^H NMR spectra were very broad, presumably due to similar steric hindrance and restricted rotation to that revealed by the X-ray structure of **14**. The situation was significantly improved by running the spectra at 55 °C, as illustrated in the case of **36** (see Supplementary Material), and the ^13^C NMR spectra for all four new ylides were run at this temperature. The value of the two-bond coupling between C=O and P was 5–7 Hz in each case, indicating a good prospect for successful thermal extrusion of Ph_3_PO [14].

### 2.3. Flash Vacuum Pyrolysis to Give Heterocyclic Products

**Scheme 6:**
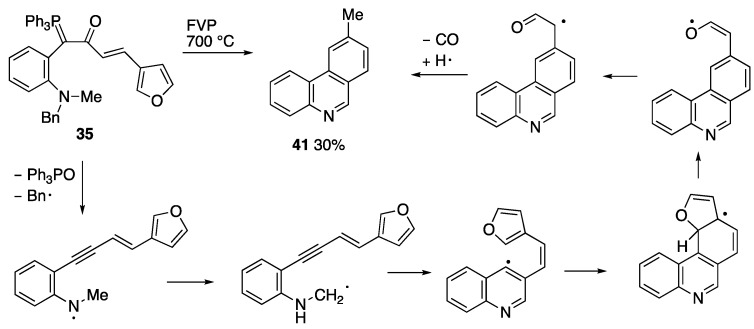
Flash vacuum pyrolysis (FVP) of ylide **35** to form **41**.

The first ylide to be pyrolyzed, the 3-furyl compound **35**, gave a surprising result. There was complete reaction at a furnace temperature of 700 °C with formation of Ph_3_PO and bibenzyl—as expected—but the sole heterocyclic product identified in low yield did not contain a furan ring at all and was identified as the 9-methylphenanthridine **41**, by comparison of its ^1^H NMR data with literature values. A suggested mechanism for formation of this product (Scheme 6) involves initial extrusion of Ph_3_PO and benzyl radical followed by transfer of the reactive site from N to C, by means of a hydrogen atom transfer. Cyclization of the NH-CH_2_ onto the triple bond, aromatization, and further cyclization gives an intermediate that could very easily lose a hydrogen atom to form the expected furo[3,2-*k*]phenanthridine. However, this is apparently not favorable, and instead, ring-opening of the dihydrofuran ring, followed by rearrangement and loss of carbon monoxide leads to the observed stable methylphenanthridine product.

**Scheme 7:**
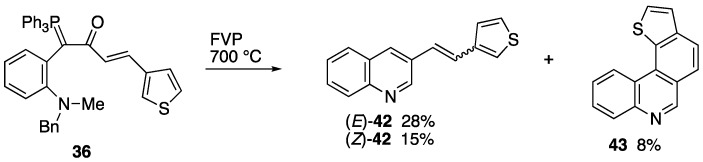
FVP of ylide **36** to form **42** and **43**.

By way of contrast, the 3-thienyl ylide **36** behaved analogously to the *N*,*N*-dibenzyl 2-thienyl ylide **25** [13] with mainly quinoline products **42**, accompanied by a small amount of the tetracyclic thieno[3,2-*k*]phenanthridine **43** formed by a further cyclization event (Scheme 7). The latter product showed a distinctive ^1^H NMR spectrum (Supplementary Material S22) which, though complex, was successfully analyzed to derive all coupling constants using simulation. Both chemical shifts and coupling constants were in good agreement with those already reported for the isomeric system **28 [13]**, thus supporting the structural assignment. Clearly, in this case, the process initially involves extrusion of Ph_3_PO and benzyl radical, hydrogen atom transfer, and cyclization, as shown in Scheme 6—but the greater thermodynamic stability of the thiophene ring means that it survives to appear in the products. The contrast between the behaviour shown here for **35** and **36** and that for the corresponding 2-furyl and 2-thienyl isomers **17** and **18,** which give exclusively fused-ring carbazole products **20** and **21** [12], is most surprising. The balance between the *N*- and *C*-centered radical as the cyclizing species is obviously delicate.

**Scheme 8:**
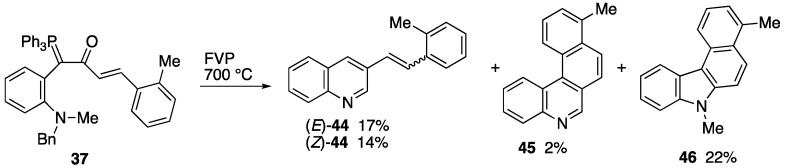
FVP of ylide **37** to form **44**–**46**.

This fact was emphasized by the result in the case of the 2-methylstyryl ylide **37** (Scheme 8). This is the methyl analogue of **16** which gave the benzocarbazole product **19** as the only major product in 66% yield [12]. Although products derived from cyclization of a *C*-centered radical, the quinolines **44** and the benzophenanthridine **45**, amounted together to 33%, the largest single product was now the dimethylbenzocarbazole **46**, resulting from direct cyclization of an *N*-centered radical, formed in 22% yield.

**Scheme 9:**
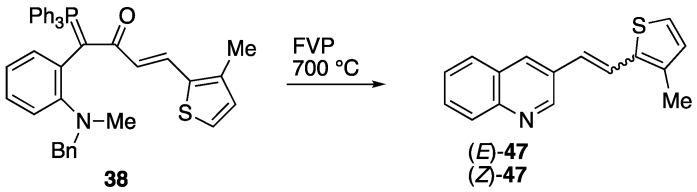
FVP of ylide **38** to form **47**.

As mentioned in the introduction, one reason for trying ylide **37** was that in earlier work we observed the involvement of an *ortho*-methyl group in such ylide pyrolyses to give naphthalenes [16,17]. This was not observed for **37** nor for the second methyl-containing ylide **38,** which simply afforded the (*E*)- and (*Z*)-thienylvinylquinolines **47,** corresponding to **42** in low yield (Scheme 9). However, even in this case, the contrast with the behavior of the ylide **18** caused by introduction of the single methyl group remote from the reactive site for initial cyclization is remarkable.

## 3. Experimental

### 3.1. General Experimental Details

NMR spectra were recorded on solutions in CDCl_3_ using Bruker instruments, and chemical shifts are given in ppm to high frequency from Me_4_Si (H, C) or 85% H_3_PO_4_ (P) with coupling constants *J* in Hz. Both low- and high-resolution mass spectra were recorded using electrospray ionization. All chromatographic separation was carried out on silica gel.

Flash vacuum pyrolysis (FVP) was carried out in a conventional flow system by subliming the starting material through a horizontal quartz tube (30 × 2.5 cm), externally heated by a tube furnace to 700 °C and maintained at a pressure of 2–3 × 10^−2^ torr by a rotary vacuum pump. The apparatus used is illustrated, and a detailed experimental procedure is given in a recent publication [19]. Products were collected in a liquid N_2_-cooled, U-shaped trap, and purified as noted.

The phosphonium salt **39** was prepared as previously described [12]. The α,β-unsaturated carboxylic acids were prepared from the corresponding aldehydes by a standard Doebner reaction with malonic acid in pyridine with catalytic piperidine.

### 3.2. X-ray Structure Determination for ***14***

The compound was prepared as previously reported [12] and recrystallized from ethyl acetate/diethyl ether (1:1) to give crystals suitable for X-ray diffraction.

Crystal data for C_40_H_34_NO_3_PS, *M* = 639.71 g·mol^−1^, yellow prism, crystal dimensions 0.10 × 0.10 × 0.10 mm, monoclinic, space group *P*2_1_/n, *a* = 10.055(2), *b* = 18.021(3), *c* = 18.125(4) A, *β* = 99.928(5)°, *V* = 3234.9(11) A^3^, *Z* = 4, *D*_calc_ = 1.313 g·cm^−3^, *T* = 93(2) K, *R* = 0.033, *R_w_* = 0.079 for 5185 reflections with *I* > 2*σ*(*I*) and 418 variables. Data were collected using graphite-monochromated Mo Kα radiation *λ* = 0.71073 A and have been deposited at the Cambridge Crystallographic Data Centre as CCDC 1849881. The data can be obtained free of charge from the Cambridge Crystallographic Data Centre via www.ccdc.cam.ac.uk/getstructures.

### 3.3. Preparation of N-Acylbenzotriazoles

#### 3.3.1. 1-(3-(3-Furyl)propenoyl)benzotriazole **31**

To a stirred solution of benzotriazole (17.15 g, 143.9 mmol) in CH_2_Cl_2_ (200 mL), thionyl chloride (4.28 g, 2.61 mL, 35.9 mmol) was added dropwise. After stirring the resulting mixture for 30 min, 3-(3-furyl)acrylic acid [20] (5.00 g, 35.9 mmol) was added and the mixture stirred at rt for a further 3 h. The mixture was then filtered and the solid washed with CH_2_Cl_2_. The combined filtrate was washed with 2M aq. NaOH water, then brine. Drying and evaporation, followed by recrystallization of the residue (CH_2_Cl_2_), gave **31** (3.25 g, 38%) as colorless needles, m.p. 169–170 °C. (Found: M^+^ + Na, 262.0598. C_13_H_9_NaN_3_O_2_ (M^+^ + Na) requires, 262.0592); *v*_max_/cm^−1^ 1703, 1621 (CO), 1379, 1151 and 992; δ_H_ 8.41 (1 H, d, *J* 8, H-7), 8.15 (1 H, d, *J* 8, H-4), 8.06 (1 H, d, *J* 16, COCH=CH), 7.87–7.79 (2 H, m, COCH=CH, furyl-H), 7.68 (1 H, t, *J* 8, H-5 or 6), 7.56–7.51 (2 H, m, H-5 or 6, furyl-H) and 6.83 (1 H, d, *J* 2, H-4 of furyl); δ_C_ 163.9 (4ry, CO), 146.4 (CH), 146.2 (4ry, C-3a of Bt), 144.8 (CH), 138.6 (C-2 of furyl), 131.4 (4ry, C-7a of Bt), 130.2 (CH), 126.1 (CH), 123.0 (4ry, C-3 of furyl), 120.1 (CH), 115.7 (CH), 114.7 (CH), and 107.6 (C-4 of furyl); *m*/*z* (ES^+^) 262.04 (M^+^ + Na, 100%).

#### 3.3.2. 1-(3-(3-Thienyl)propenoyl)benzotriazole **32**

This was prepared as in Section 3.3.1 using benzotriazole (15.25 g, 128.0 mmol), thionyl chloride (3.81 g, 2.32 mL, 32.4 mmol), and 3-(3-thienyl)acrylic acid (5.00 g, 32.4 mmol). Drying and evaporation, followed by recrystallization of the residue (CH_2_Cl_2_), gave **32** (5.30 g, 65%) as yellow needles, m.p. 154–156 °C. (Found: M^+^ + Na, 278.0370. C_13_H_9_NaN_3_OS (M^+^ + Na) requires, 278.0364); *v*_max_/cm^−1^ 1696 (CO), 1611, 1285, 1069, 993, 782 and 743; δ_H_ 8.42 (1 H, dt, *J* 8, 1, H-7), 8.15 (1 H, dt, *J* 8, 1, H-4), 8.14 (1 H, d, *J* 15, COCH=CH), 7.93 (1 H, d, *J* 15, COCH=CH), 7.76 (1 H, dd, *J* 3, 1, H-2 of thienyl), 7.68 (1 H, td, *J* 8, 1, H-5 or 6), 7.55 (1 H, dd, *J* 5, 1, H-5 of thienyl), 7.53 (1 H, td, *J* 8, 1, H-5 or 6) and 7.43 (1 H, ddd, *J* 5, 3, 1, H-4 of thienyl); δ_C_ 164.0 (4ry, CO), 146.1 (4ry, C-3a of Bt), 141.7 (CH), 137.5 (4ry, C-7a of Bt), 131.3 (4ry, C-3 of thienyl), 130.7 (CH), 130.1 (CH), 127.3 (CH), 126.0 (CH), 125.4 (CH), 120.0 (CH), 115.4 (CH) and 114.7 (CH); *m*/*z* (ES^+^) 277.98 (M^+^ + Na, 100%).

#### 3.3.3. 1-(3-(2-Methylphenyl)propenoyl)benzotriazole **33**

This was prepared as in Section 3.3.1 using benzotriazole (14.69 g, 123.3 mmol), thionyl chloride (3.67 g, 2.24 mL, 30.8 mmol) and (*E*)-3-(2-methylphenyl)prop-2-enoic acid (5.00 g, 30.8 mmol). Drying and evaporation, followed by recrystallization of the residue (CH_2_Cl_2_), gave **33** (1.77 g, 22%) as a white powder, m.p. 124–125 °C (Lit. [21] 127–129 °C); δ_H_ 8.47 (1 H, d, *J* 15, COCH=CH), 8.42 (1 H, dt, *J* 8, 1, H-4 or 7 of Bt), 8.16 (1 H, dt, *J* 8, 1, H-4 or 7 of Bt), 8.07 (1 H, d, *J* 15, COCH=CH), 7.86 (1 H, d, *J* 8), 7.69 (1 H, ddd, *J* 8, 7, 1, H-5 or 6 of Bt), 7.54 (1 H, ddd, *J* 8, 7, 1, H-5 or 6 of Bt), 7.40–7.27 (3 H, m) and 2.56 (3 H, s, Me).

#### 3.3.4. 1-(3-(3-Methyl-2-thienyl)propenoyl)benzotriazole **34**

This was prepared as in Section 3.3.1 using benzotriazole (14.16 g, 118.9 mmol), thionyl chloride (3.53 g, 2.16 mL, 79.7 mmol), and (*E*)-3-(3-methyl-2-thienyl)prop-2-enoic acid (5.00 g, 29.7 mmol). Drying and evaporation, followed by recrystallization of the residue (CH_2_Cl_2_), gave **34** (4.20 g, 60%) as pale yellow powder, m.p. 169–170 °C. (Found: M^+^ + Na, 292.0521. C_14_H_11_NaN_3_OS (M^+^ *+* Na) requires, 292.0521); *v*_max_/cm^−1^ 1699 (CO), 1601, 1380, 1278 and 988; δ_H_ 8.39 (1 H, dt, *J* 8, 1, H-4 or 7 of Bt), 8.32 (1 H, dd, *J* 16, 1, COCH=CH), 8.14 (1 H, dt, *J* 8, 1, H-4 or 7 of Bt), 7.80 (1 H, d, *J* 16, COCH=CH), 7.67 (1 H, ddd, *J* 8, 7, 1, H-5 or 6 of Bt), 7.52 (1 H, ddd, *J* 8, 7, 1, H-5 or 6 of Bt), 7.42 (1 H, br d, *J* 5), 6.94 (1 H, dd, *J* 5, 0.3) and 2.46 (3 H, s, Me); δ_C_ 169.9 (4ry, CO), 146.2 (4ry), 144.0 (2C, 4ry), 139.2 (CH), 134.0 (4ry), 131.4 (CH), 130.1 (CH), 129.2 (CH), 126.0 (CH), 120.1 (CH), 114.7 (CH), 113.2 (CH), and 14.4 (CH_3_); *m*/*z* (ES^+^) 292.01 (M^+^ + Na, 100%).

### 3.4. Preparation of Ylides

#### 3.4.1. [(2-(*N-*Methyl-*N-*benzylamino)phenyl)(3-(3-furyl)propenoyl)methylene]triphenylphosphorane **35**

A suspension of the phosphonium salt **39** (2.00 g, 3.62 mmol) in dry THF (10 mL) was stirred under nitrogen while a solution of butyllithium in hexanes (1.60 cm^3^, 2.25 M, 3.62 mmol) was added. The resulting red solution of **40** was stirred at rt for 2 h, after which a solution of the *N-*acylbenzotriazole **31** (0.86 g, 3.62 mmol) in THF (5 mL) was added and the mixture stirred for a further 18 h. Water (20 mL) was then added, and the resulting mixture extracted with ethyl acetate (2 × 20 mL). The combined extracts were washed with water, dried, and evaporated. The resulting solid was recrystallized (Et_2_O/EtOAc) to give **35** (0.89 g, 41%) as yellow crystals, m.p. 184–185 °C; (Found: M^+^ + H, 592.2411. C_40_H_35_NO_2_P (M^+^ + H), requires 592.2405); ν_max_/cm^−1^ 1633, 1434, 1105, 970, 748 and 692; δ_H_ 7.87–7.27 (15 H, m), 7.21–7.09 (6 H, m), 7.07–6.79 (7 H, m), 6.40 (1 H, d, *J* 8, H-3 of Ar), 4.45 (1 H, d, *J* 14, CHHPh), 3.75 (1 H, d, *J* 14, CHHPh) and 2.10 (3 H, s, Me); δ_C_ (+55 °C) 178.9 (d, *J* 6, C=O), 154.1 (d, *J* 4, ArC-N), 143.3 (furyl C-O), 142.7 (furyl C-O), 138.4 (d, *J* 5, CH), 136.9 (C), 133.8 (d, *J* 10, C-2 of PPh), 131.8 (d, *J* 10, P=C-C), 131.4 (d, *J* 2, C-4 of PPh), 129.5 (2CH), 128.3 (d, *J* 12, C-3 of PPh), 127.8 (2CH), 127.7 (d, *J* 3), 126.9 (CH), 126.7 (d, *J* 91, C-1 of PPh), 126.3 (d, *J* 12, CH), 124.7 (CH), 124.1 (furyl C-3), 122.8 (d, *J* 2, CH), 121.3 (d, *J* 2, CH), 107.9 (furyl C-4), 75.3 (d, *J* 107, P=C), 60.1 (CH_2_), and 39.6 (NMe); δ_P_ + 15.3; *m*/*z* (ES^+^) 592.09 (M^+^ + H, 100%).

#### 3.4.2. [(2-(*N-*Methyl-*N-*benzylamino)phenyl)(3-(3-thienyl)propenoyl)methylene]triphenylphosphorane **36**

This was prepared as in Section 3.4.1 using salt **39** (1.35 g, 2.44 mmol), a solution of butyllithium (1.08 cm^3^, 2.25 M, 2.44 mmol) and *N-*acylbenzotriazole **32** (0.55 g, 2.44 mmol). The resulting solid was recrystallized (Et_2_O/EtOAc) to give **36** (0.51 g, 35%) as orange crystals, m.p. 178–180 °C. (HMRS: found M^+^ + H, 608.2175. C_40_H_35_NOPS (M^+^ + H) requires, 608.2177); *v*_max_/cm^−1^ 1624, 1492, 1197, 1101, 750 and 691; δ_H_ (+55 °C) 7.68–7.54 (8 H, m), 7.45–7.33 (5 H, m), 7.30–7.21 (4 H, m), 7.19–7.11 (5 H, m), 7.09–7.02 (2 H, m), 6.99–6.89 (4 H, m), 6.52 (1 H, d, *J* 8), 4.40 (1 H, d, *J* 14, CHHPh), 3.84 (1 H, d, *J* 14, CHHPh) and 2.19 (3 H, s, Me); δ_C_ (+55 °C) 179.1 (d, *J* 5, C=O), 154.2 (d, *J* 4, ArC-N), 139.8 (thienyl C-3), 138.4 (d, *J* 5, CH), 137.0 (C), 133.8 (d, *J* 10, C-2 of PPh), 131.8 (d, *J* 10, P=C-C), 131.4 (d, *J* 3, C-4 of PPh), 129.5 (2CH), 129.1 (d, *J* 1, CH), 128.3 (d, *J* 12, C-3 of PPh), 127.8 (2CH), 127.8 (d, *J* 3), 126.9 (CH), 126.7 (d, *J* 91, C-1 of PPh), 126.4 (d, *J* 12, CH), 126.0 (CH), 125.6 (CH), 124.6 (CH), 122.8 (d, *J* 2, CH), 121.3 (d, *J* 2, CH), 75.7 (d, *J* 108, P=C), 60.1 (CH_2_), and 39.6 (NMe); δ_P_ + 15.3; *m*/*z* (ES^+^) 608.01 (M^+^ + H, 100%).

#### 3.4.3. [(2-(*N-*Methyl-*N-*benzylamino)phenyl)(3-(2-methyl)phenylpropenoyl)methylene]triphenylphosphorane **37**

This was prepared as in Section 3.4.1 using salt **39** (1.00 g, 1.81 mmol), a solution of butyllithium in hexanes (0.89 mL, 2.04 M, 1.81 mmol) and *N-*acylbenzotriazole **33** (0.48 g, 1.81 mmol). Recrystallization of the residue (diethyl ether/EtOAc) gave the title product **37** (0.55 g, 50%) as yellow crystals, m.p. 207–208 °C. (Found: M^+^ + H, 616.2753. C_43_H_39_NOP (M^+^ + H) requires, 616.2769); ν_max_/cm^−1^ 1634, 1433, 1172, 1086, 710 and 677; δ_H_ 7.75 (1 H, d, *J* 15, CH = CH), 7.70–7.59 (5 H, br s), 7.51–7.31 (11 H, m), 7.24–7.16 (3 H, m), 7.12–6.93 (9 H, m), 6.55 (1 H, d, *J* 8, H-3 of Ar), 4.51 (1 H, d, *J* 14, CHHPh), 3.85 ( 1 H, d, *J* 14, CHHPh), 2.39 (3 H, s, Me) and 2.24 (3 H, s, Me); δ_C_ (+55 °C) 179.7 (d, *J* 7, C=O), 154.3 (d, *J* 4, ArC-N), 138.8 (d, *J* 5, CH), 137.3 (C), 137.0 (C), 136.3 (C), 133.8 (d, *J* 10, C-2 of PPh), 132.3 (d, *J* 10, P=C-C), 132.2 (CH), 131.1 (d, *J* 3, C-4 of PPh), 130.3 (CH), 129.5 (2CH), 128.5 (d, *J* 12, CH), 128.4 (CH), 128.2 (d, *J* 12, C-3 of PPh), 127.8 (2CH), 127.6 (CH), 127.4 (d, *J* 90, C-1 of PPh), 126.8 (CH), 126.3 (CH), 125.7 (CH), 122.6 (d, *J* 2, CH), 121.0 (d, *J* 2, CH), 72.8 (d, *J* 109, P=C), 60.0 (CH_2_), 39.5 (NMe), and 20.0 (Me); δ_P_ + 15.4; *m*/*z* (ES^+^) 616.08 (M^+^ + H, 100%).

#### 3.4.4. [(2-(*N-*Methyl-*N-*benzylamino)phenyl)(3-(3-methyl-2-thienyl)propenoyl)methylene]triphenylphosphorane **38**

This was prepared as in Section 3.4.1 using salt **39** (1.00 g, 1.81 mmol), a solution of butyllithium in hexanes (0.89 mL, 2.04 M, 1.81 mmol) and *N-*acylbenzotriazole **34** (0.49 g, 1.81 mmol). The resulting solid was recrystallized (Et_2_O/EtOAc) to give the title product **38** (0.55 g, 49%) as orange crystals, m.p. 197–198 °C. (Found: M^+^ + H, 622.2347. C_41_H_37_NOPS (M^+^ + H) requires, 622.2333); *v*_max_/cm^−1^ 1720, 1605, 1196, 1094, 719 and 686; δ_H_ (+55 °C) 7.71–7.51 (7 H, br m), 7.48–7.27 (10 H, m), 7.19–7.14 (3 H, m), 7.05–6.88 (5 H, m), 6.81 (1 H, d, *J* 15, P=C), 6.70 (1 H, d, *J* 5), 6.44 (1 H, d, *J* 8, C-3 of Ar), 4.45 (1 H, d, *J* 14, CHHPh), 3.72 (1 H, d, *J* 14, CHHPh), 2.22 (3 H, s, Me) and 2.15 (3 H, s, Me); δ_C_ (+55 °C) 179.1 (d, *J* 6, C=O), 153.9 (d, *J* 4, ArC-N), 138.5 (d, *J* 5, CH), 136.8 (C), 136.5 (d, *J* 1, C), 133.8 (d, *J* 10, C-2 of PPh), 132.1 (d, *J* 10, P=C-C), 131.2 (d, *J* 2, C-4 of PPh), 130.7 (CH), 129.8 (2CH), 128.4 (d, *J* 12, CH), 128.2 (d, *J* 12, C-3 of PPh), 127.7 (2CH), 127.4 (d, *J* 2), 127.2 (d, *J* 92, C-1 of PPh), 126.7 (CH), 126.1 (d, *J* 1, CH), 126.0 (C), 123.6 (CH), 122.6 (CH), 121.3 (d, *J* 2, CH), 72.9 (d, *J* 108, P=C), 60.0 (CH_2_), 39.3 (NMe), and 14.0 (Me); δ_P_ + 15.7; *m*/*z* (ES^+^) 622.03 (M^+^ + H, 100%).

### 3.5. Flash Vacuum Pyrolysis of Ylides

#### 3.5.1. FVP of Ylide **35**

Ylide **35** (0.3234 g, 0.54 mmol) was subjected to FVP at 700 °C and 2–3 × 10^−2^ torr. NMR analysis of the crude product showed a mixture of Ph_3_PO, bibenzyl, and other products. Separation by column chromatography (9:1 diethyl ether:hexane) followed by preparative TLC (60:40 diethyl ether:hexane) gave 9-methylphenanthridine **41** (0.031 g, 30%), δ_H_ 9.25 (1 H, s, H-6), 8.59 (1 H, ddd, *J* 8, 1, 0.4, H-1), 8.42 (1 H, d, *J* 1, H-10), 8.20 (1 H, dd, *J* 8, 1, H-8), 8.00 (1 H, d, *J* 8, H-7), 7.75 (1 H, tdd, *J* 8, 1, 0.4, H-2 or 3), 7.68 (1 H, tdd, *J* 8, 1, 0.4, H-2 or 3), 7.56 (1 H, ddd, *J* 8, 1, 0.4, H-4) and 2.68 (3 H, ArMe) [Lit. [22] δ_H_ (DMSO) 9.05 (1 H, s), 8.28 (1 H, dd, *J* 8.2, 1.3), 8.12 (1 H, dd, *J* 8.1, 1.2), 7.64–7.58 (2 H, m), 7.52–7.46 (1 H, m), 7.23 (1 H, dd, *J* 8.1, 1.2), and 2.41 (3 H, s).]; *m*/*z* (ES^+^) 194.09 (M^+^ + H, 10%).

#### 3.5.2. FVP of Ylide **36**

Ylide **36** (0.2147 g, 0.35 mmol) was subjected to FVP at 700 °C and 2–3 × 10^−2^ torr. NMR analysis of the crude product showed a mixture of Ph_3_PO, bibenzyl, and other products. The mixture was separated by column chromatography (60:40 diethyl ether:hexane) giving 3-(2-(3-thienyl)ethenyl)quinoline **42** (*E*, 0.0235 g, 28%. *Z*, 0.0126 g, 15%); (Found: M^+^ + H, 238.0689. C_15_H_12_NS (M^+^+H) requires, 238.0690); δ_H_ (*E*) 9.09 (1 H, d, *J* 2, H-2), 8.14 (1 H, d, *J* 2, H-4), 8.09 (1 H, ddd, *J* 8, 1.2, 0.6, H-8), 7.81 (1 H, ddd, *J* 8, 1.2, 0.6, H-5), 7.67 (1 H, ddd, *J* 8, 7, 1.2, H-6 or 7), 7.54 (1 H, ddd, *J* 8, 7, 1.2, H-6 or 7), 7.43 (1 H, m, H-4′), 7.38–7.35 (2 H, m, H-2′ and 5′), and 7.35 and 7.09 (2 H, AB pattern, *J* 16, H-a and b); δ_H_ (*Z*) 8.82 (1 H, d, *J*-2, H-2), 8.05 (1 H, d, *J* 2, H-4), 8.07 (1 H, ddd, *J* 8, 1.2, 0.6, H-8), 7.73 (1 H, ddd, *J* 8, 1.2, 0.6, H-5), 7.69 (1 H, ddd, *J* 8, 6, 1.2, H-6 or 7), 7.53 (1 H, ddd, *J* 8, 7, 1.2, H-6 or 7), 7.18–7.15 (2 H, m, H-2′ and 5′), 6.86 (1 H, ddd, *J* 3, 2.5, 1, H-4′) and 6.76 and 6.65 (2 H, AB pattern, *J* 12, H-a and b); *m*/*z* (ES^+^) 238.01 (M^+^ + H, 100%). Also obtained was thieno[3,2-*k*]phenanthridine **43** (0.0066 g, 8%); (Found: M^+^ + H, 236.0535. C_15_H_10_NS (M^+^ + H) requires 236.0534); δ_H_ 9.46 (1 H, s, H-6), 9.09 (1 H, ddd, *J* 9.2, 1.2, 0.8, H-11), 8.39 (1 H, ddd, *J* 9.2, 1.2, 0.8, H-8), 8.22 (1 H, d, *J* 8, H-4 or 5), 8.08 (1 H, d, *J* 8, H-4 or 5), 7.91 (1 H, d, *J* 5.6, H-2 or 3), 7.90 (1 H, ddd, *J* 9.2, 7.6, 1.2, H-9 or 10), 7.88 (1 H, ddd, *J* 9.2, 7.6, 1.2, H-9 or 10), and 7.71 (1 H, d, *J* 5.6, H-2 or 3); *m*/*z* (ES^+^) 236.04 (M^+^ + H, 100%).

#### 3.5.3. FVP of Ylide **37**

Ylide **37** (0.1971 g, 0.32 mmol) was subjected to FVP at 700 °C and 2–3 × 10^−2^ torr. NMR analysis of the crude product showed a mixture of Ph_3_PO, bibenzyl, and other products. The mixture was roughly separated into two fractions by column chromatography (50:50 diethyl ether:hexane). The first fraction was purified by preparative TLC (10:90 diethyl ether:hexane) to give 4,7-dimethylbenzo[c]carbazole **46** (0.0179 g, 22%); (Found: M^+^ + H, 246.1279. C_18_H_16_N (M^+^ + H) requires, 246.1283); δ_H_ 8.71 (1 H, ddd, *J* 8.4, 0.8, 0.5, H-1), 8.605 (1 H, ddd, *J* 8, 1.2, 0.5, H-11), 8.119 (1 H, dd, *J* 9.2, 0.5, H-5), 7.696 (1 H, d, *J* 9.2, H-6), 7.602 (1 H, dd, *J* 8.4, 6.8, H-2), 7.562 (1 H, ddd, *J* 8, 1.2, 0.5, H-8), 7.515 (1 H, ddd, *J* 8, 7.2, 1.2, H-9), 7.385 (1 H, ddd, *J* 8, 7.2, 1.2, H-10), 7.324 (1 H, dd, *J* 6.8, 0.8, H-3), 4.00 (3H, s, NMe) and 2.82 (3H, s, ArMe); δ_C_ 130.1 (4ry), 126.5 (CH), 124.1 (CH), 124.0 (CH), 123.1 (CH), 122.2 (CH), 121.6 (CH), 120.9 (4ry), 120.3 (4ry), 119.6 (CH), 115.6 (4ry), 114.6 (4ry), 113.9 (4ry), 110.0 (CH), 109.0 (CH), 29.3 (NMe) and 20.4 (ArMe); *m*/z (ES^+^) 246.08 (M^+^ + H, 100%). The second fraction was purified by preparative TLC (10:90 diethyl ether:hexane) to give 3-(2-(2-methylphenyl)ethenyl)quinoline **44** (*E*, 0.0134 g, 17%. *Z*, 0.0101 g, 14%); (Found: M^+^ + H, 246.1291. C_18_H_16_N (M^+^ + H) requires, 246.1283); δ_H_ (*E*) 9.13 (1 H, d, *J* 2, H-2), 8.18 (1 H, d, *J* 2, H-4), 8.10 (1 H, d, *J* 8, H-5 or 8), 7.84 (1 H, dd, *J* 8, 1, H-5 or 8), 7.69 (1 H, ddd, *J* 8, 7, 1, H-6 or 7), 7.66 (1 H, d, *J* 8), 7.56 (1 H, half of AB pattern, *J* 16, H-a or b), 7.55 (1 H, ddd, *J* 8, 7, 1, H-6 or 7), 7.29–7.20 (3 H, m), 7.15 (1 H, half of AB pattern, *J* 16, H-a or b) and 2.49 (3 H, s, Me); δ_H_ (*Z*) 8.62 (1 H, d, *J* 2, H-2), 8.09 (1 H, d, *J* 8, H-5 or 6), 7.84 (1 H, d, *J* 2, H-4), 7.63 (1 H, ddd, *J* 8, 6.5, 1, H-6 or 7), 7.61 (1 H, d, *J* 8), 7.46 (1 H, ddd, *J* 8, 6.5, 1, H-6 or 7), 7.19 (1 H, br t, *J* 7.5), 7.12 (1 H, d, *J* 8), 7.04 (1 H, br t, *J* 7.5), 6.89 and 6.75 (2 H, AB pattern, *J* 12, H-a and b) and 2.32 (3 H, s, Me); *m*/*z* (ES^+^) 246.00 (M^+^ + H, 100%). Also isolated from the same preparative TLC was 9-methylbenzo[*k*]phenanthridine **45** (0.0017 g, 2%), (Found: M^+^ + H, 244.1120. C_18_H_15_N (M^+^ + H) requires, 244.1126); δ_H_ 9.37 (1 H, s, H-6), 9.07 (2 H, t, *J* 8, H-1 and 12), 8.38 (1 H, dd, *J* 8, 2, H-4), 8.25 (1 H, d, *J* 9, H-8), 8.00 (1 H, d, *J* 9, H-7), 7.81 (1 H, ddd, *J* 8, 7, 1, H-2 or 3), 7.74 (1 H, ddd, *J* 8, 7, 1, H-2 or 3), 7.67 (1 H, t, *J* 7, H-11), 7.59 (1 H, d, *J* 7, H-10), and 2.87 (3 H, s, Me); *m*/*z* (ES^+^) 244.03 (M^+^ + H, 100%).

#### 3.5.4. FVP of Ylide **38**

Ylide **38** (0.1072 g, 0.17 mmol) was subjected to FVP at 700 °C and 2–3 × 10^−2^ torr. NMR analysis of the crude product showed a mixture of Ph_3_PO, bibenzyl, and other products. The mixture was purified by column chromatography (50:50 diethyl ether:hexane) followed by preparative TLC (30:70 diethyl ether:hexane) to give a low yield of what appeared to be impure 3-(2-(3-methyl-2-thienyl)ethenyl)quinoline **47** as a dark brown oil. Indicative peaks at δ_H_ 9.09 (1 H, d, *J* 2, H-2 *E*), 8.83 (1 H, d, *J* 2, H-2 *Z*), 2.39 (3 H, s, ArMe *E* or *Z*), and 2.20 (3 H, s, ArMe, *E* or *Z*) indicated a 1:1 mixture of *E/Z*—however, further purification and analysis was not possible.

## 4. Conclusions

The results from FVP of these four ylides further emphasize the sensitivity of the outcome in these reactions on the precise combination of substituents present. As compared to the previously studied *N*-benzyl-*N*-methylamino ylides, the present compounds show an almost complete change from carbazole products derived from cyclization of N(Me)^∙^ to quinoline and phenanthridine products derived from hydrogen atom transfer and cyclization of the resulting NHCH_2_^∙^. The results are more similar to those obtained from certain *N*,*N*-dibenzyl ylides, but even then, significant differences in product distribution are caused by the slightest change in ylide structure. The difference in pyrolysis outcome between the isomeric compounds **35** and **36** and **17** and **18** is striking, as is the effect of adding a single methyl substituent remote from the reactive site, as seen going from **16** to **37** or from **18** to **38**. We conclude that, as also found in our recent synthesis of Eustifoline D using this method [13], model studies are of little value in this area, and which pyrolytic routes will predominate in a given case can only be determined by experimentation.

## Supplementary Materials

The following are available online. Figures S1–S27: NMR spectra of new compounds. Cif and check-cif files for X-ray structure of **14**.
