# Supplementary material for: Further Studies on the Pyrolytic Domino Cyclization of Stabilized Phosphonium Ylides Bearing an Ortho-Aminophenyl Group
Source: Molecules. 2018 Aug 27;23(9):2153. doi: 10.3390/molecules23092153 (PMC6225108; doi:10.3390/molecules23092153)

Further studies on the pyrolytic domino cyclization of stabilized phosphonium ylides bearing an *ortho*-aminophenyl group

R. Alan Aitken*, Lorna Murray and Alexandra M. Z. Slawin

EaStCHEM School of Chemistry, University of St Andrews, North Haugh, St Andrews, Fife, KY16 9ST, UK.

Supplementary Material

Figure

^1^H NMR and DEPTQ ^13^C NMR spectra of **31** S1, S2

^1^H NMR and DEPTQ ^13^C NMR spectra of **32** S3, S4

^1^H NMR spectrum of **33** S5

^1^H NMR and DEPTQ ^13^C NMR spectra of **34** S6, S7

^1^H NMR, ^31^P and ^13^C NMR spectra of **35** S8–S10

^1^H NMR, ^31^P and ^13^C NMR spectra of **36** S11–S13

^1^H NMR, ^31^P and ^13^C NMR spectra of **37** S14–S16

^1^H NMR, ^31^P and ^13^C NMR spectra of **38** S17–S19

^1^H NMR spectrum of **41** S20

^1^H NMR spectrum of **42** S21

^1^H NMR spectrum of **43** S22

^1^H NMR spectrum of **44** S23

^1^H NMR spectrum of **45** S24

^1^H NMR and DEPTQ ^13^C NMR spectra of **46** S25, S26

^1^H NMR spectrum of **47** S27

Figure S1. 400 MHz ^1^H NMR spectrum of **31**


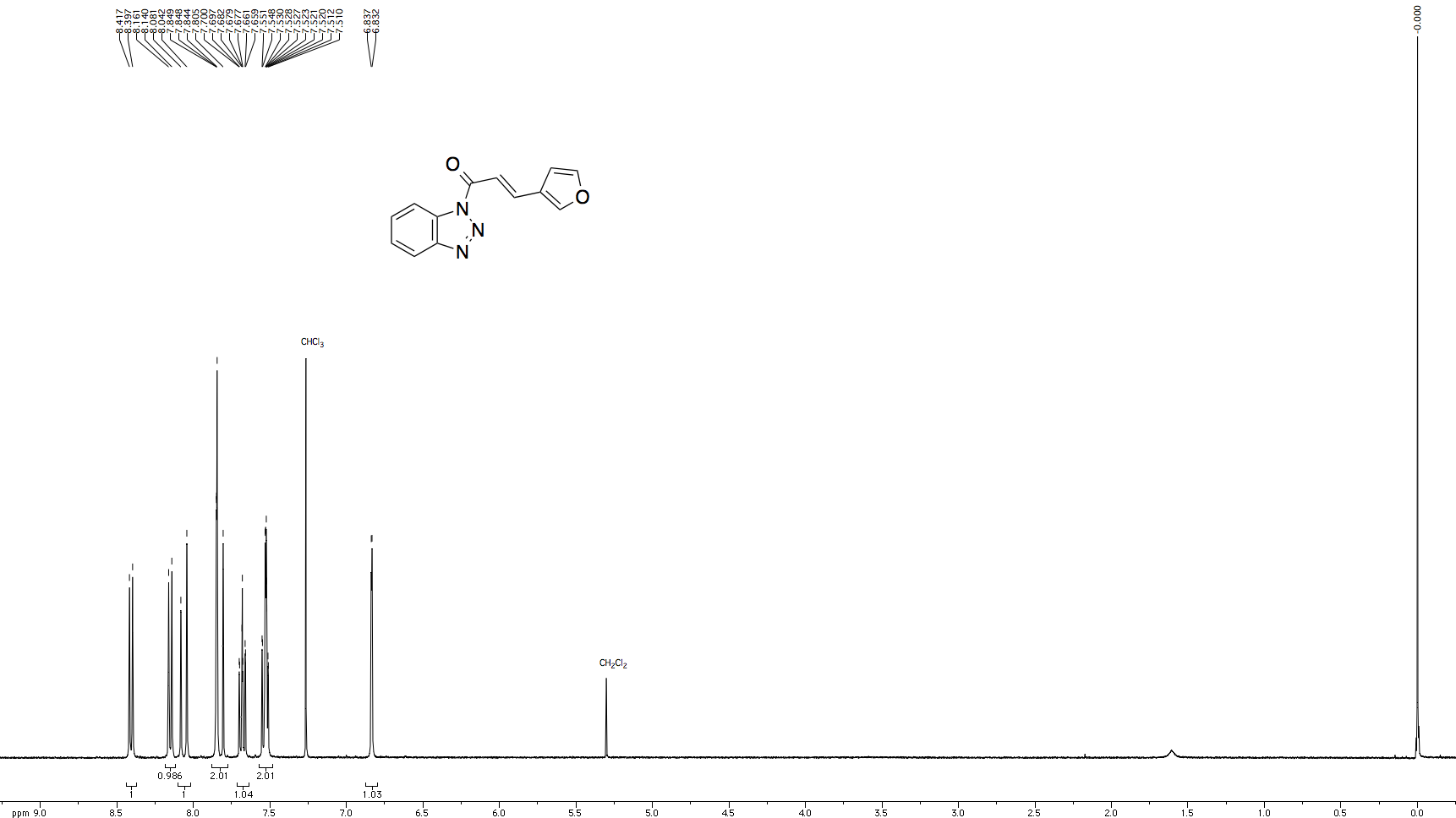


Figure S2. 75 MHz DEPTQ ^13^C NMR spectrum of **31**


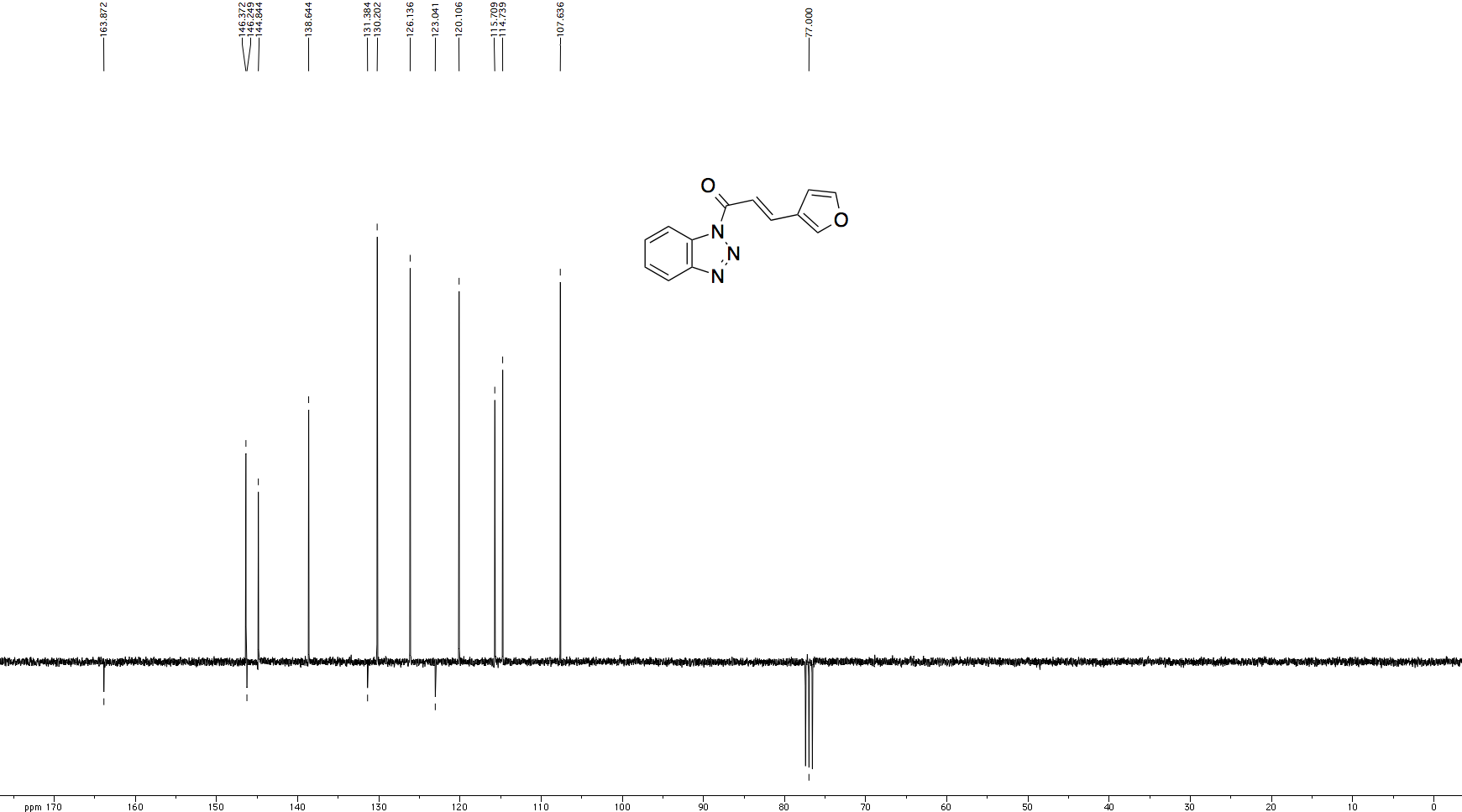


Figure S3. 400 MHz ^1^H NMR spectrum of **32**


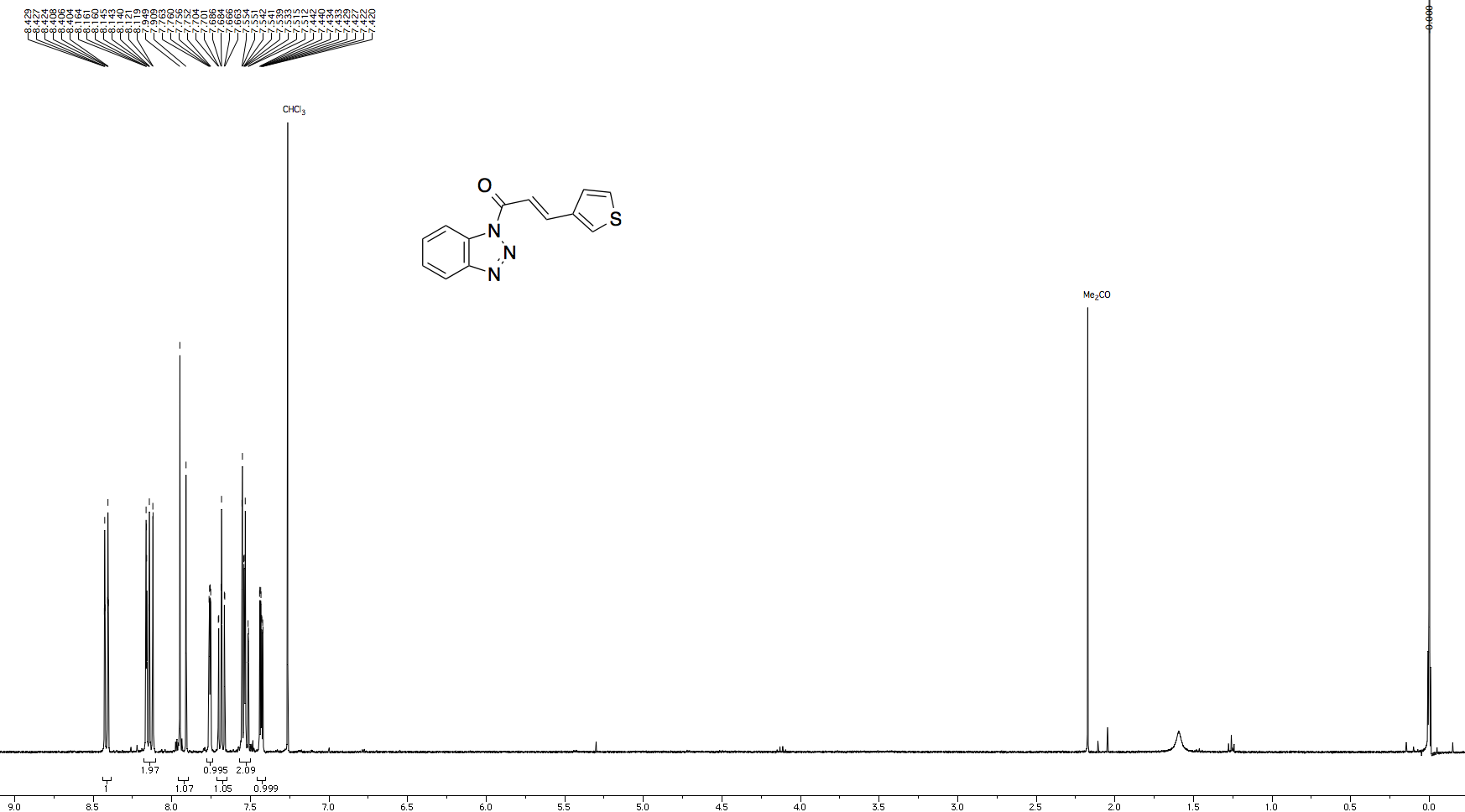


Figure S4. 75 MHz DEPTQ ^13^C NMR spectrum of **32**


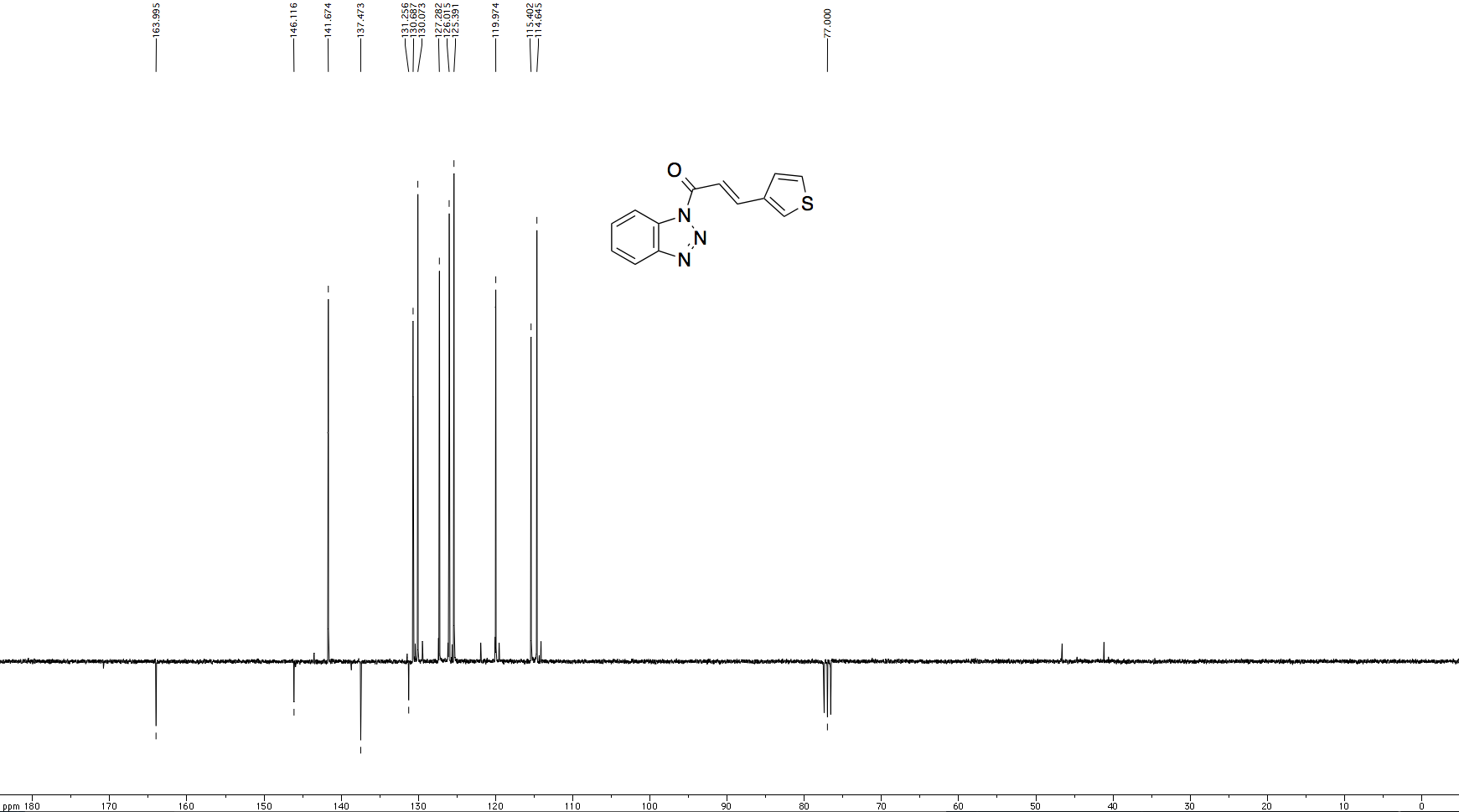


Figure S5. 300 MHz ^1^H NMR spectrum of **33**


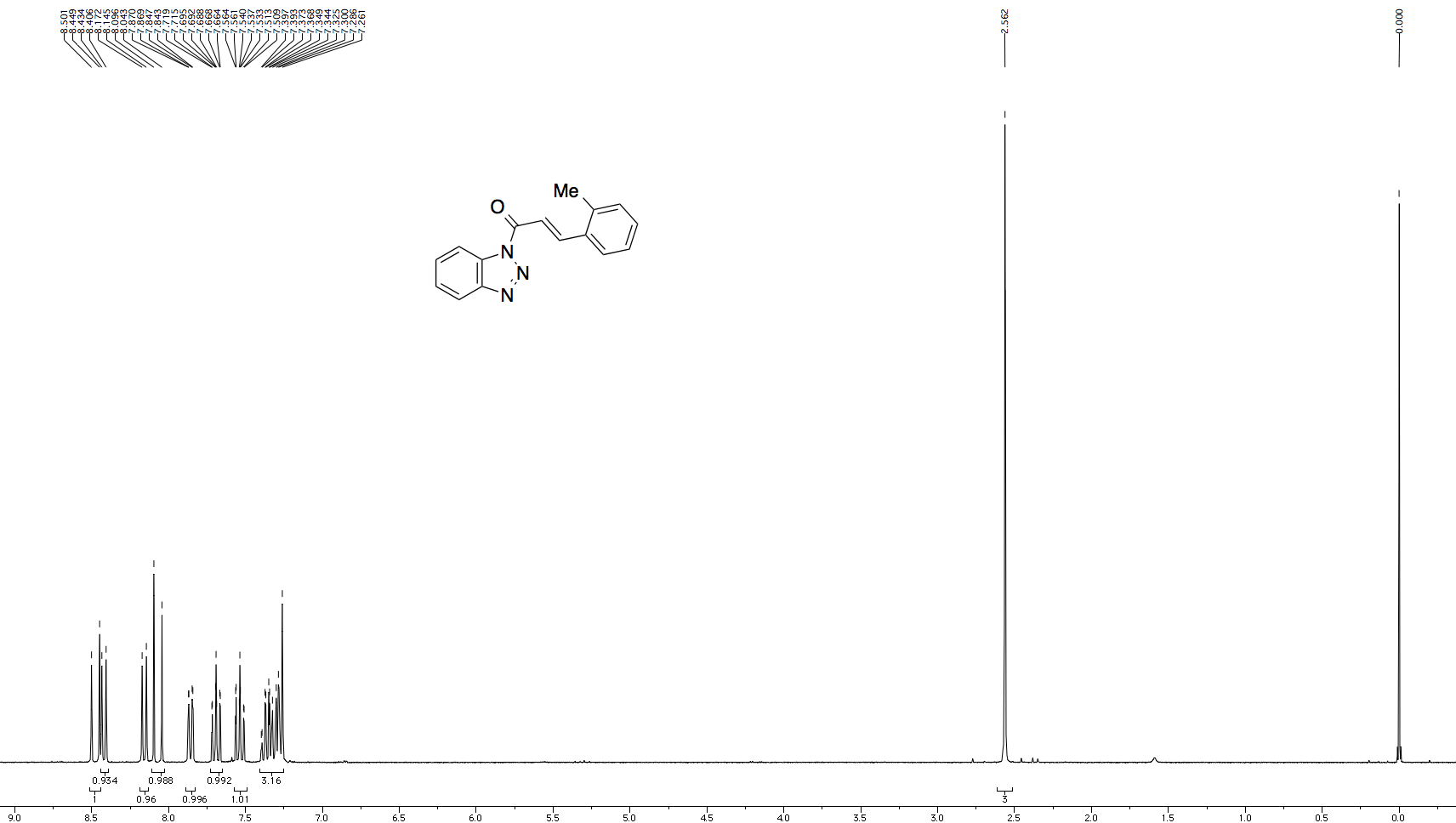


Figure S6. 300 MHz ^1^H NMR spectrum of **34**


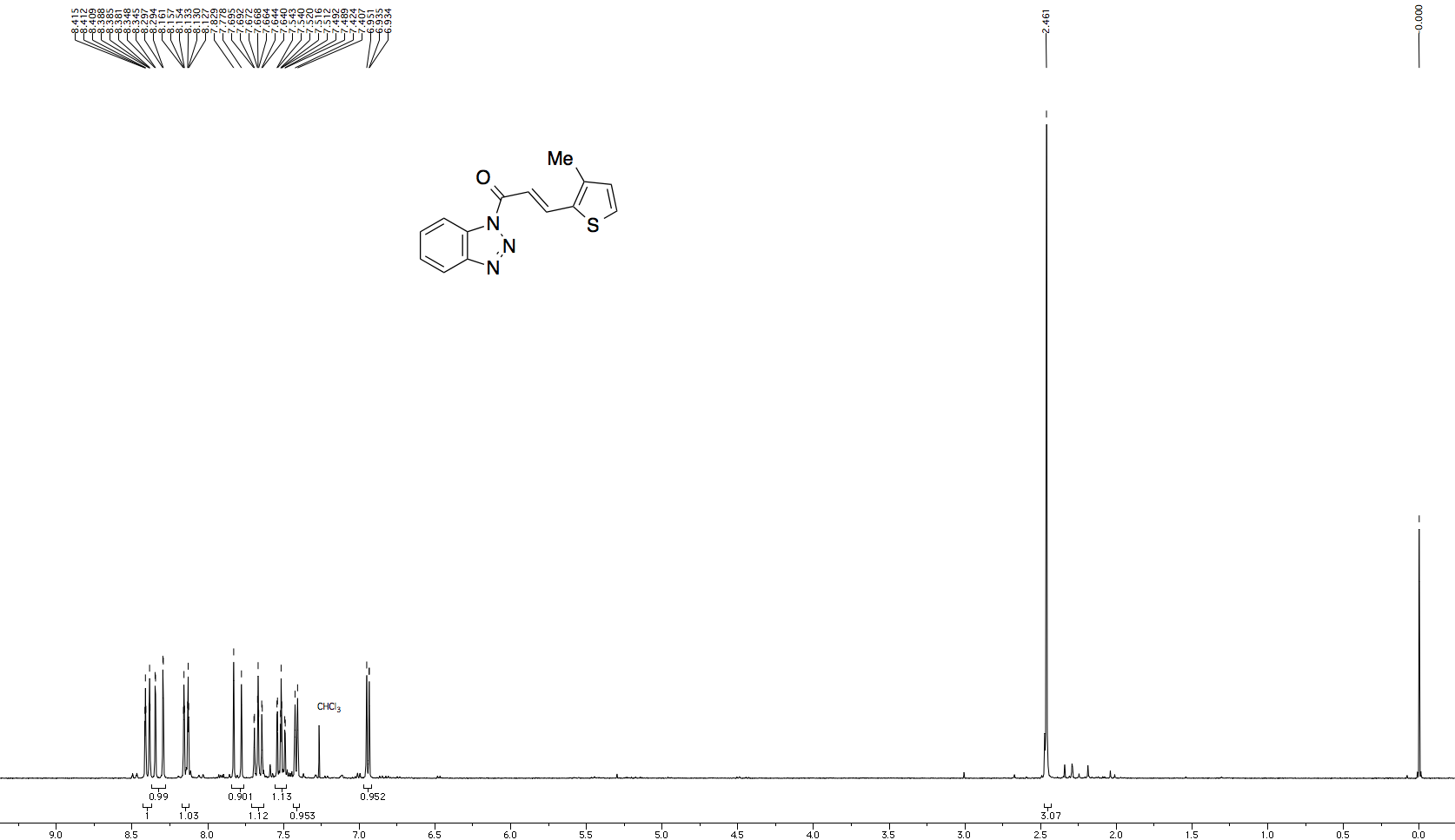


Figure S7. 75 MHz DEPTQ ^13^C NMR spectrum of **34**


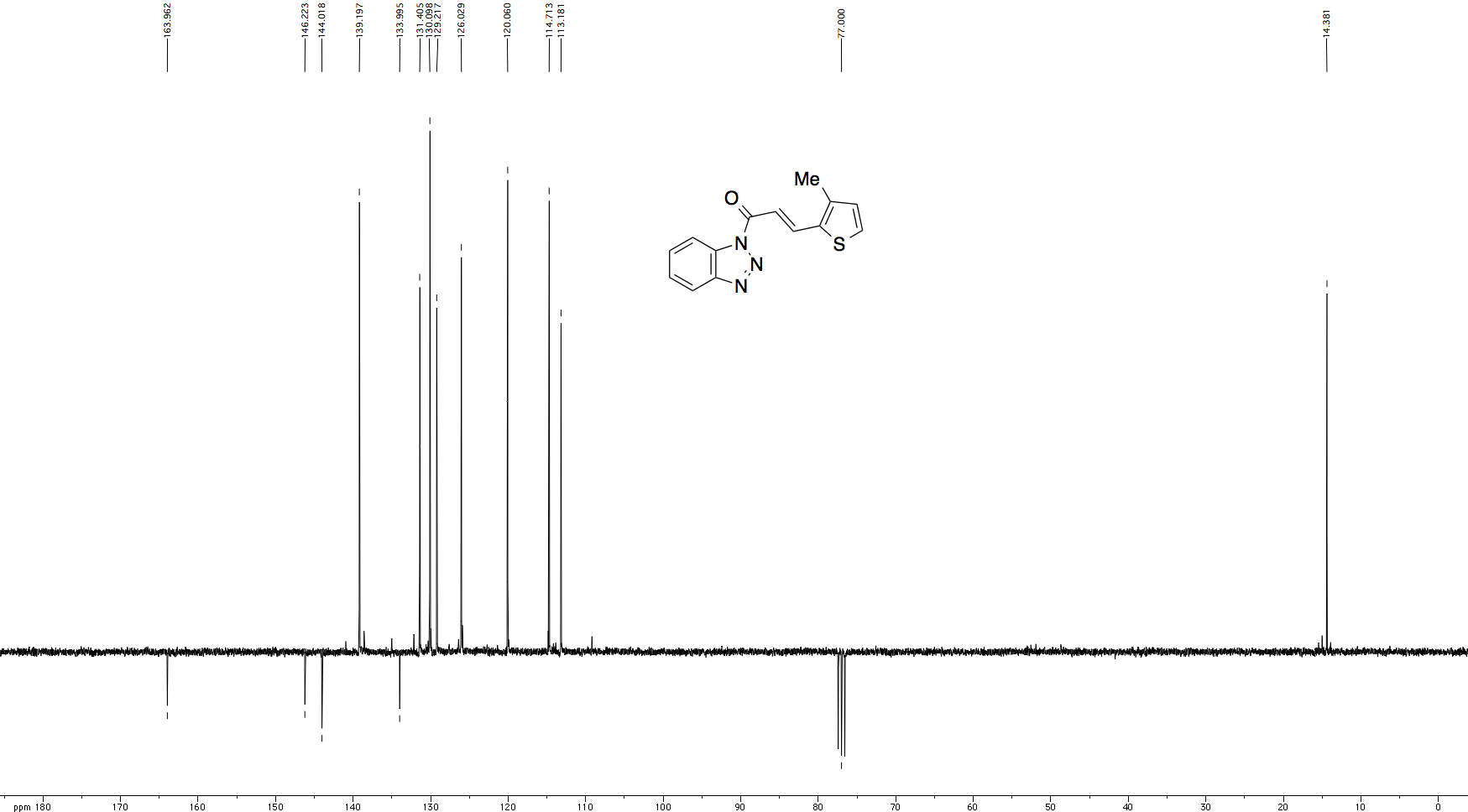


Figure S8. 300 MHz ^1^H NMR spectrum of **35**


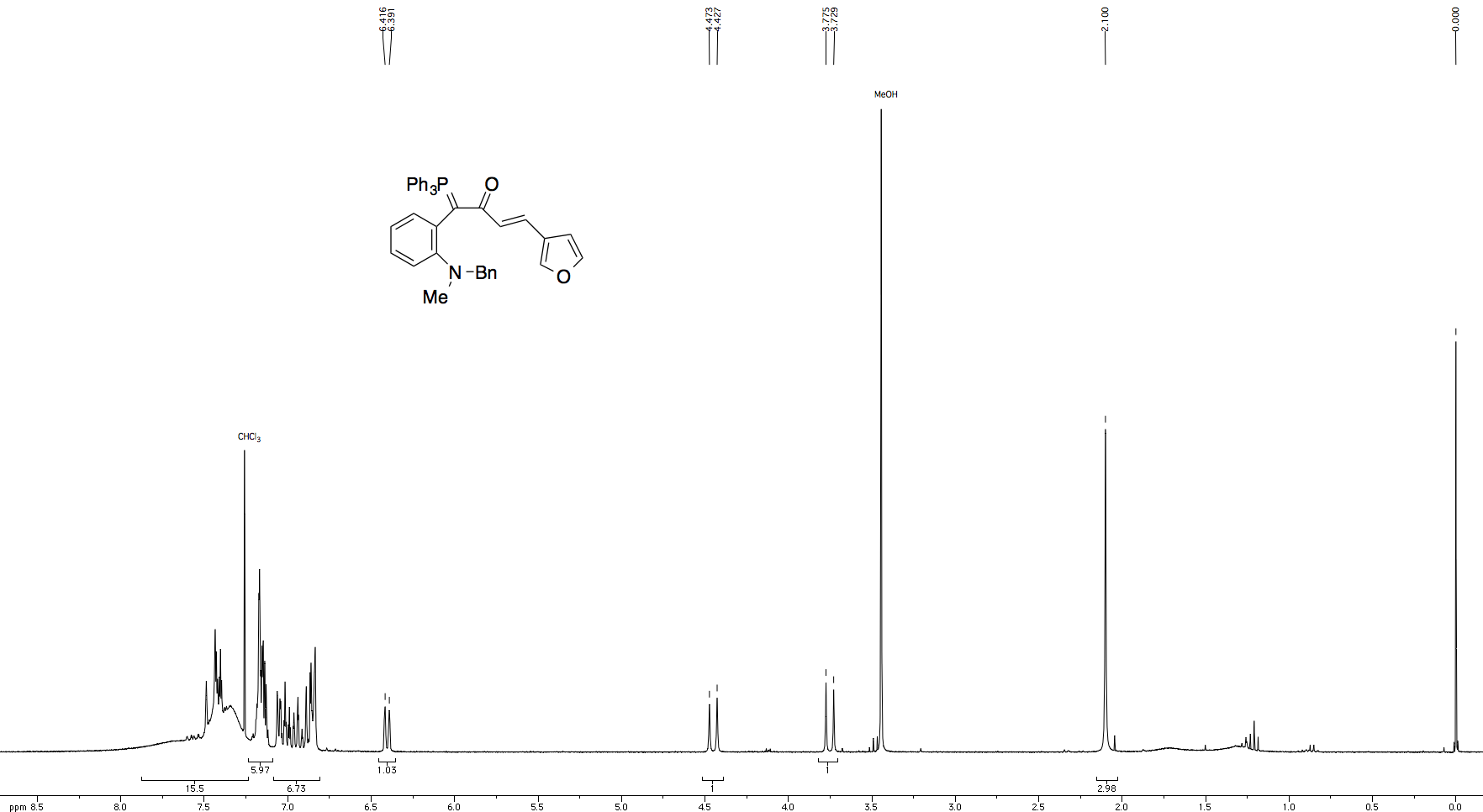


Figure S9. 121 MHz ^31^P NMR spectrum of **35**


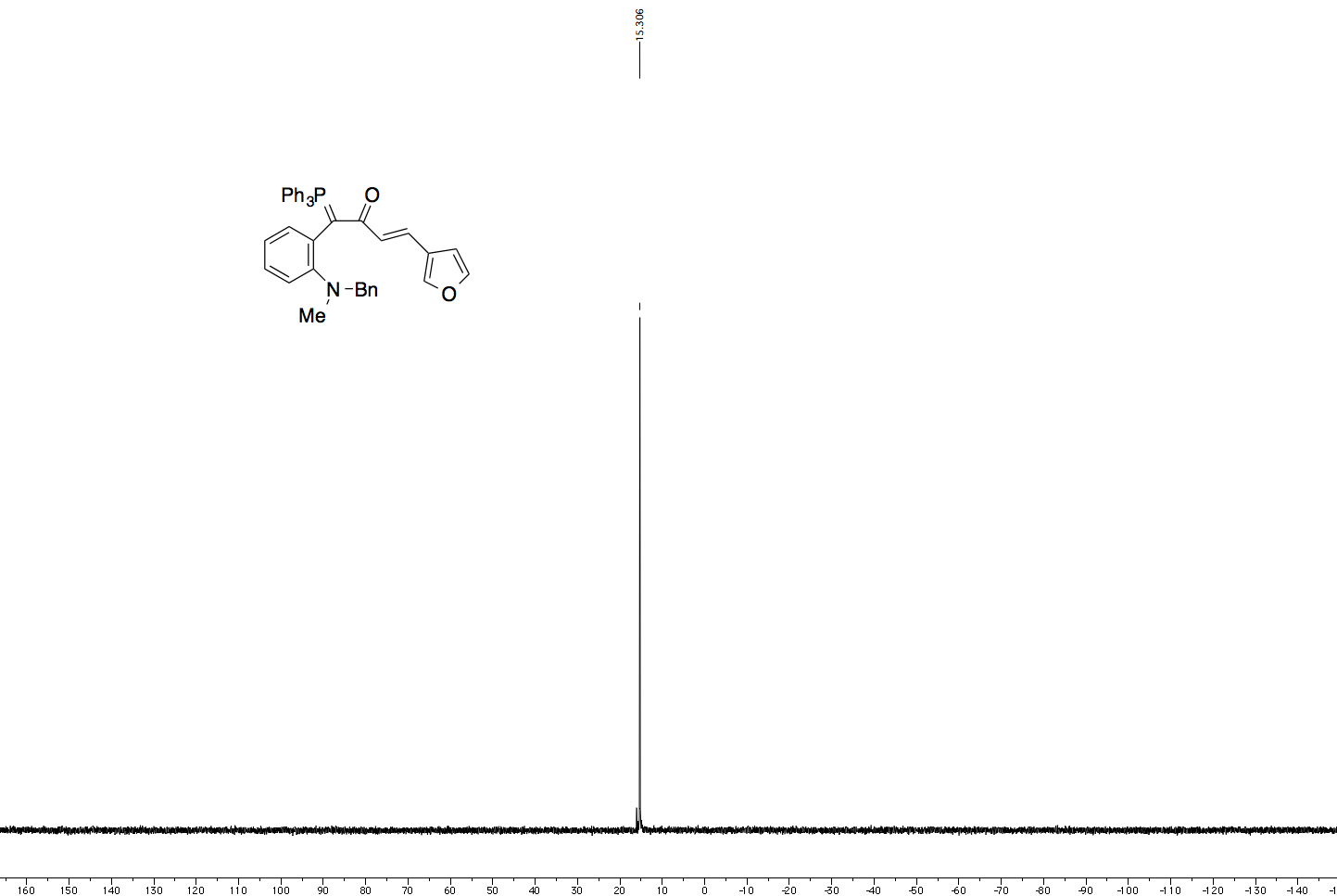


Figure S10. 75 MHz ^13^C NMR spectrum of **35** (+55 °C)


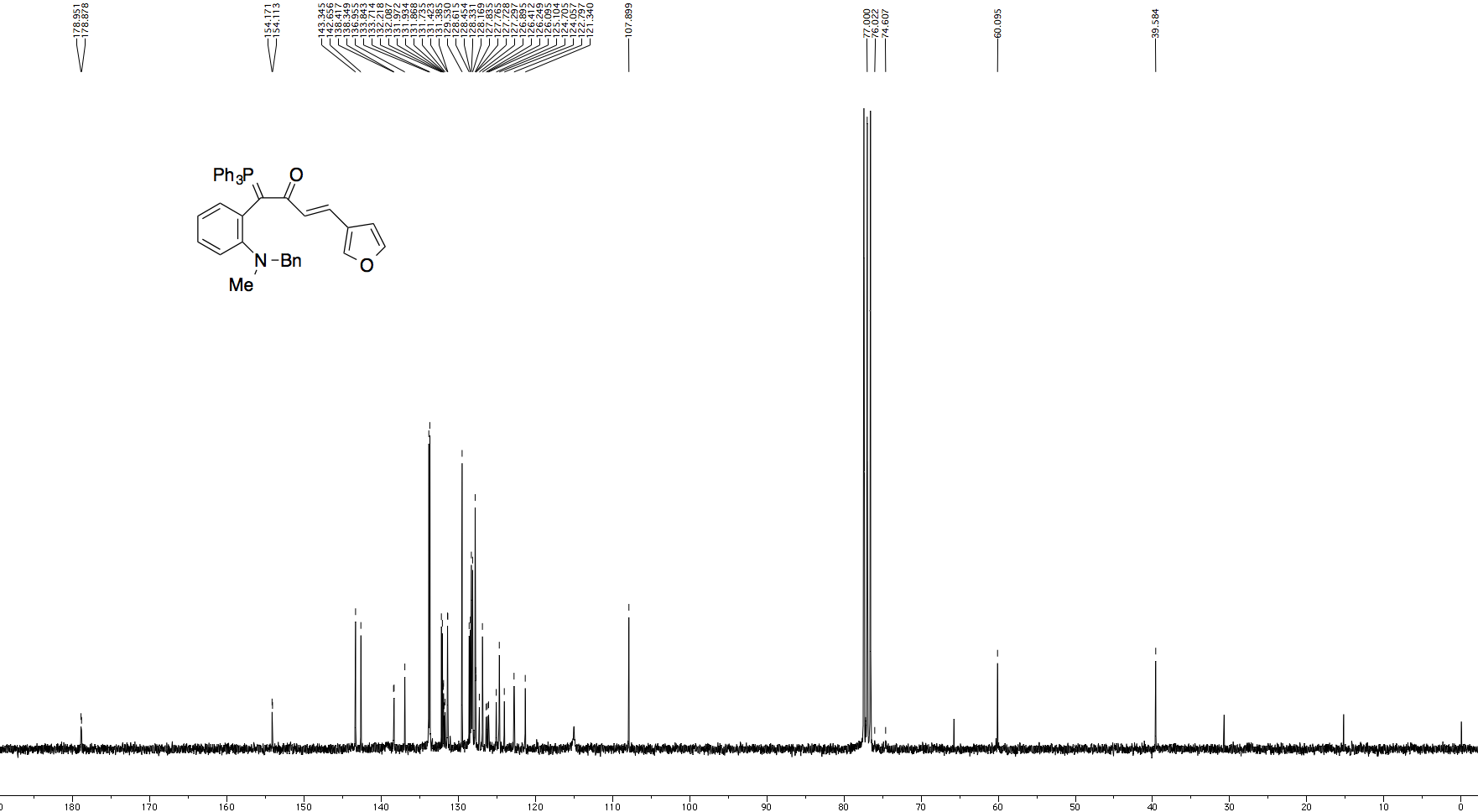


Figure S11. 300 MHz ^1^H NMR spectrum of **36** (+55 °C)


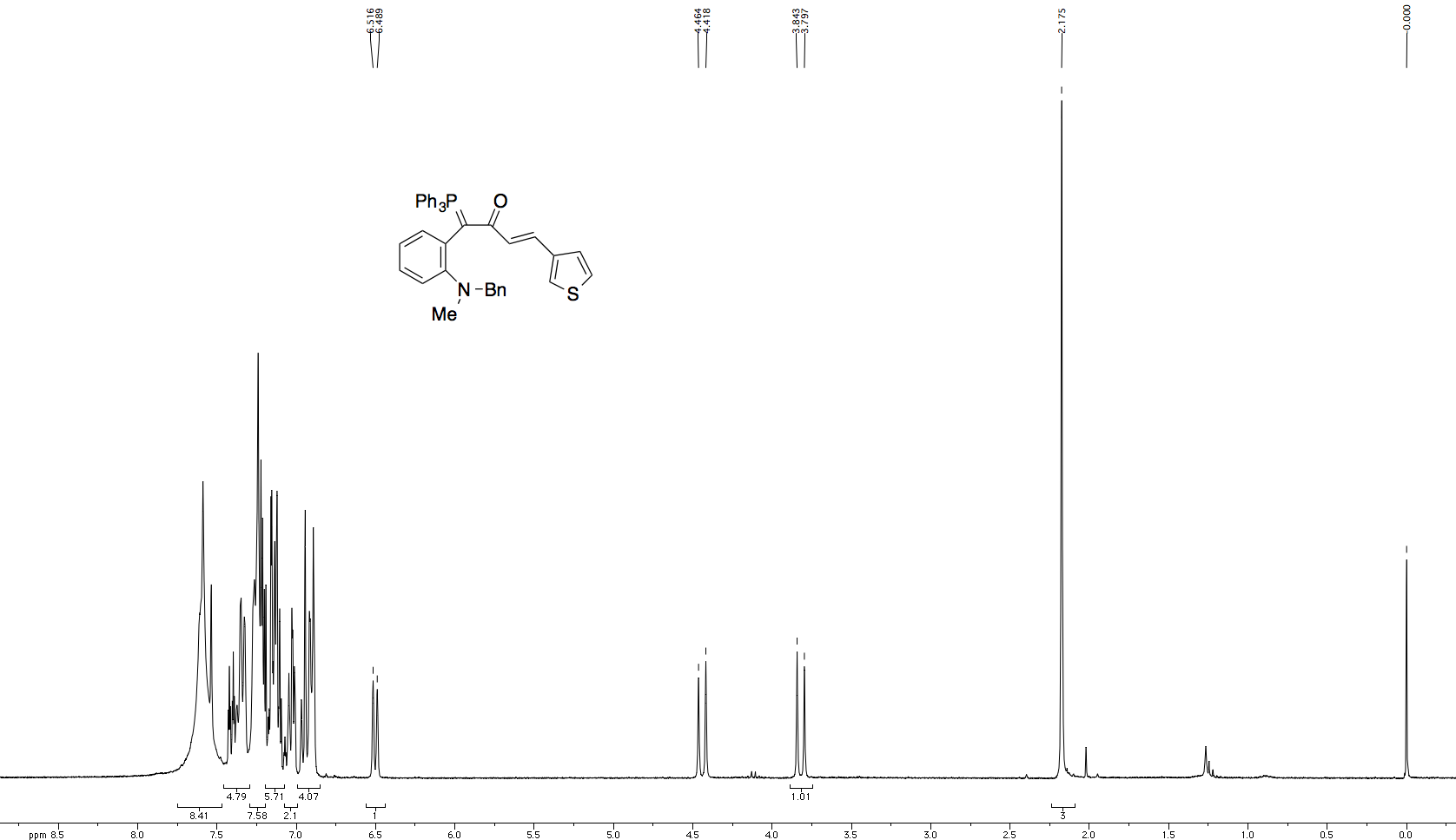


Figure S12. 161 MHz ^31^P NMR spectrum of **36**


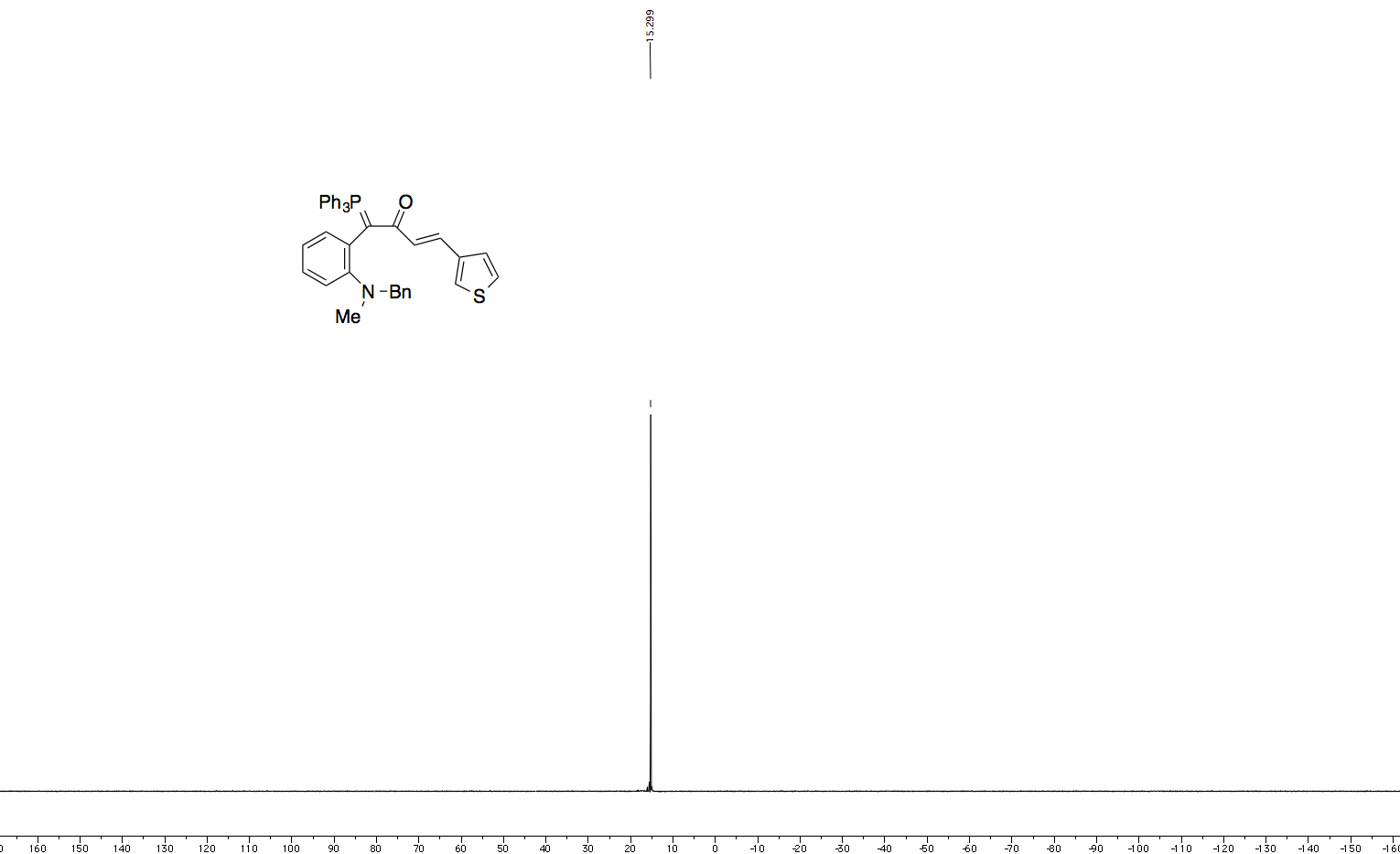


Figure S13. 75 MHz ^13^C NMR spectrum of **36** (+55 °C)


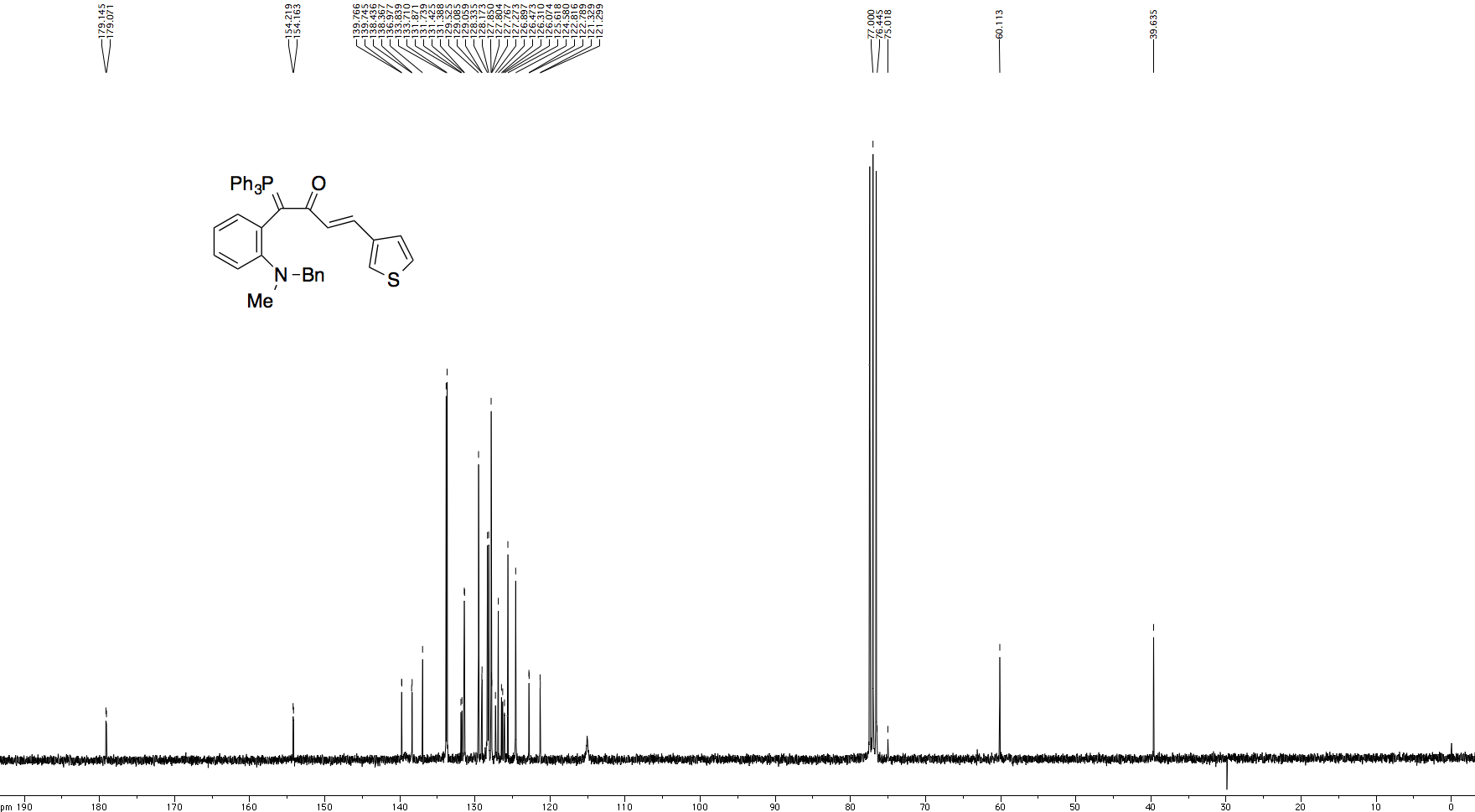


Figure S14. 300 MHz ^1^H NMR spectrum of **37**


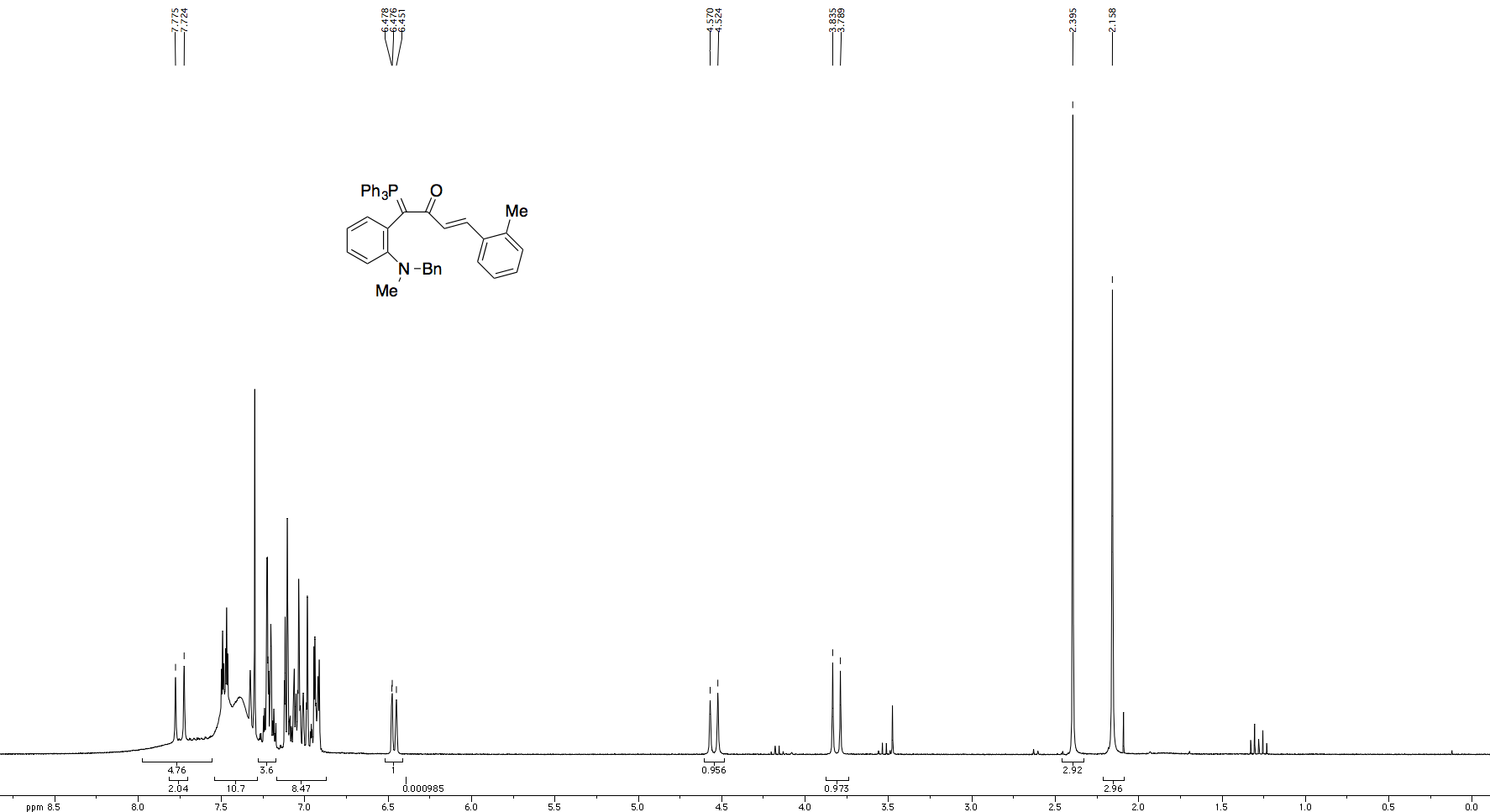


Figure S15. 121 MHz ^31^P NMR spectrum of **37**


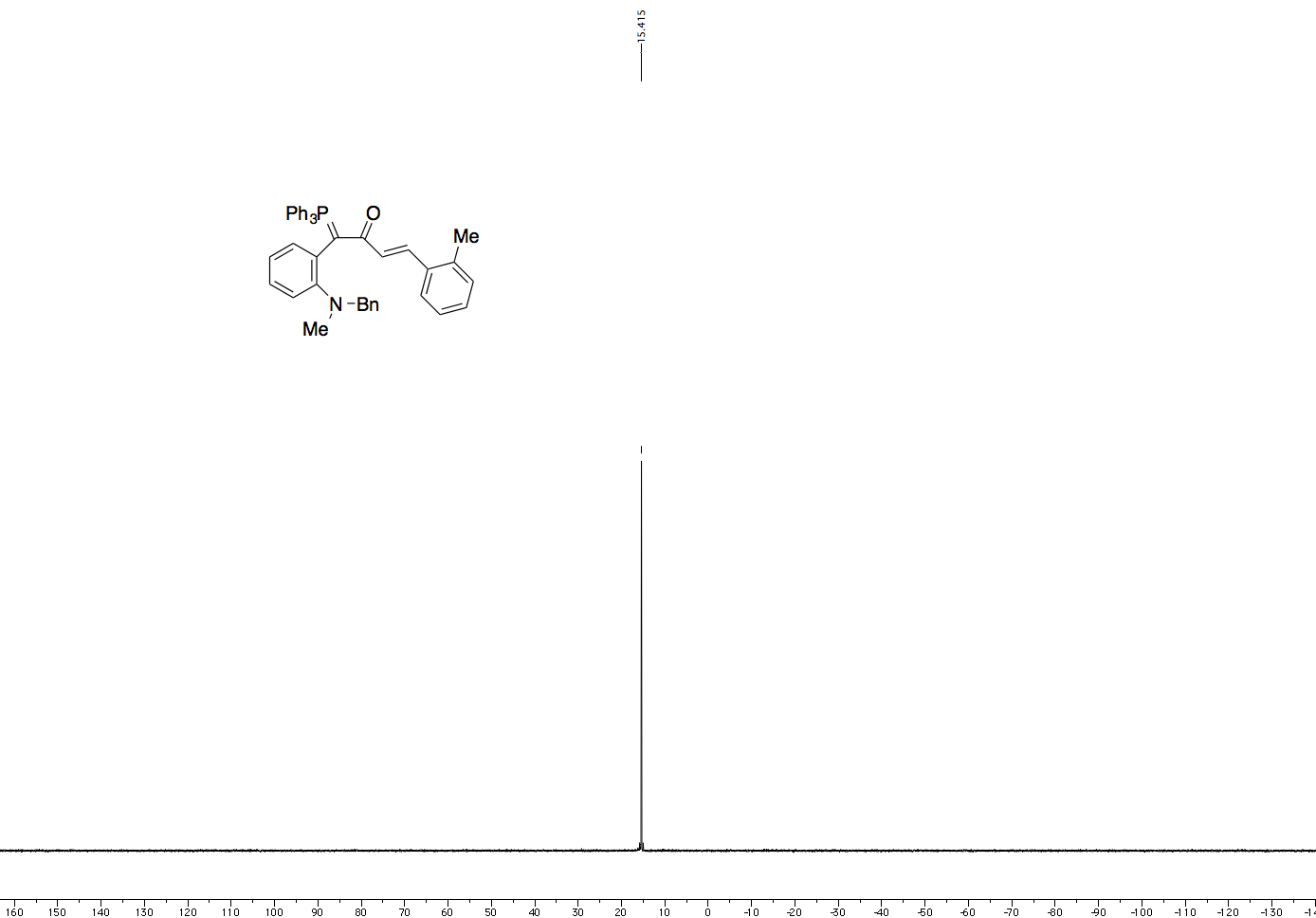


Figure S16. 75 MHz ^13^C NMR spectrum of **37** (+55 °C)


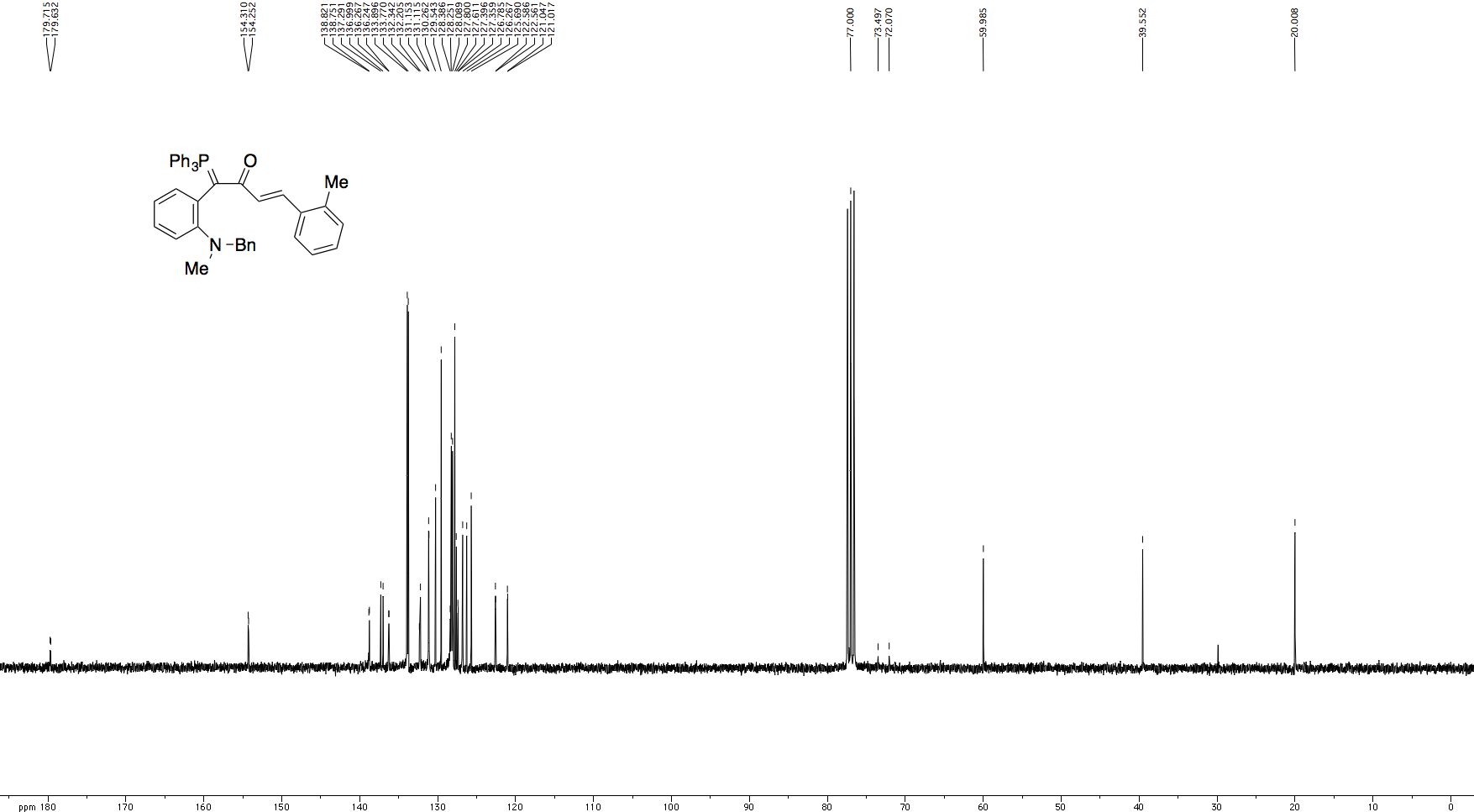


Figure S17. 300 MHz ^1^H NMR spectrum of **38**


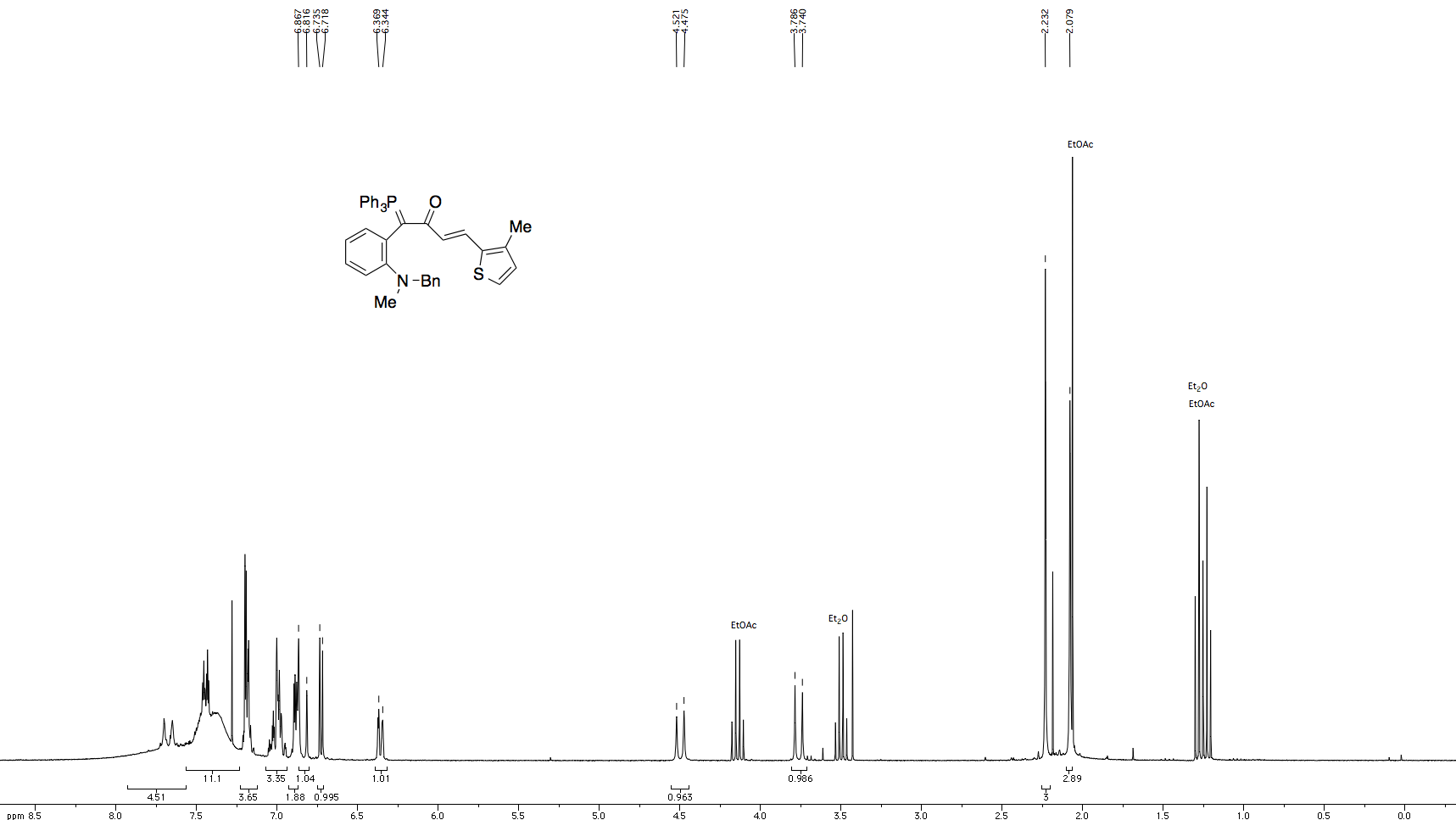


Figure S18. 121 MHz ^31^P NMR spectrum of **38**


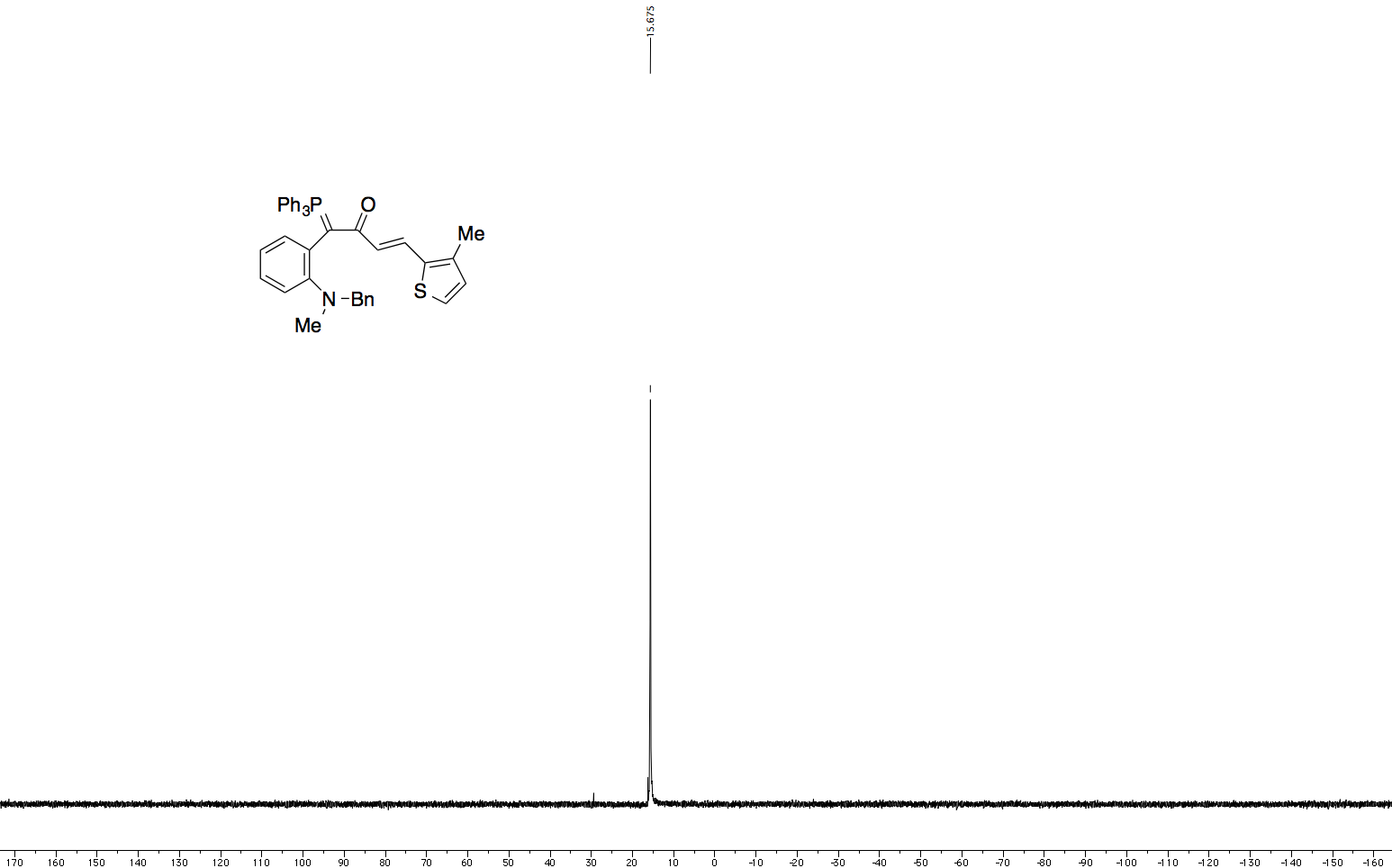


Figure S19. 75 MHz ^13^C NMR spectrum of **38** (+55 °C)


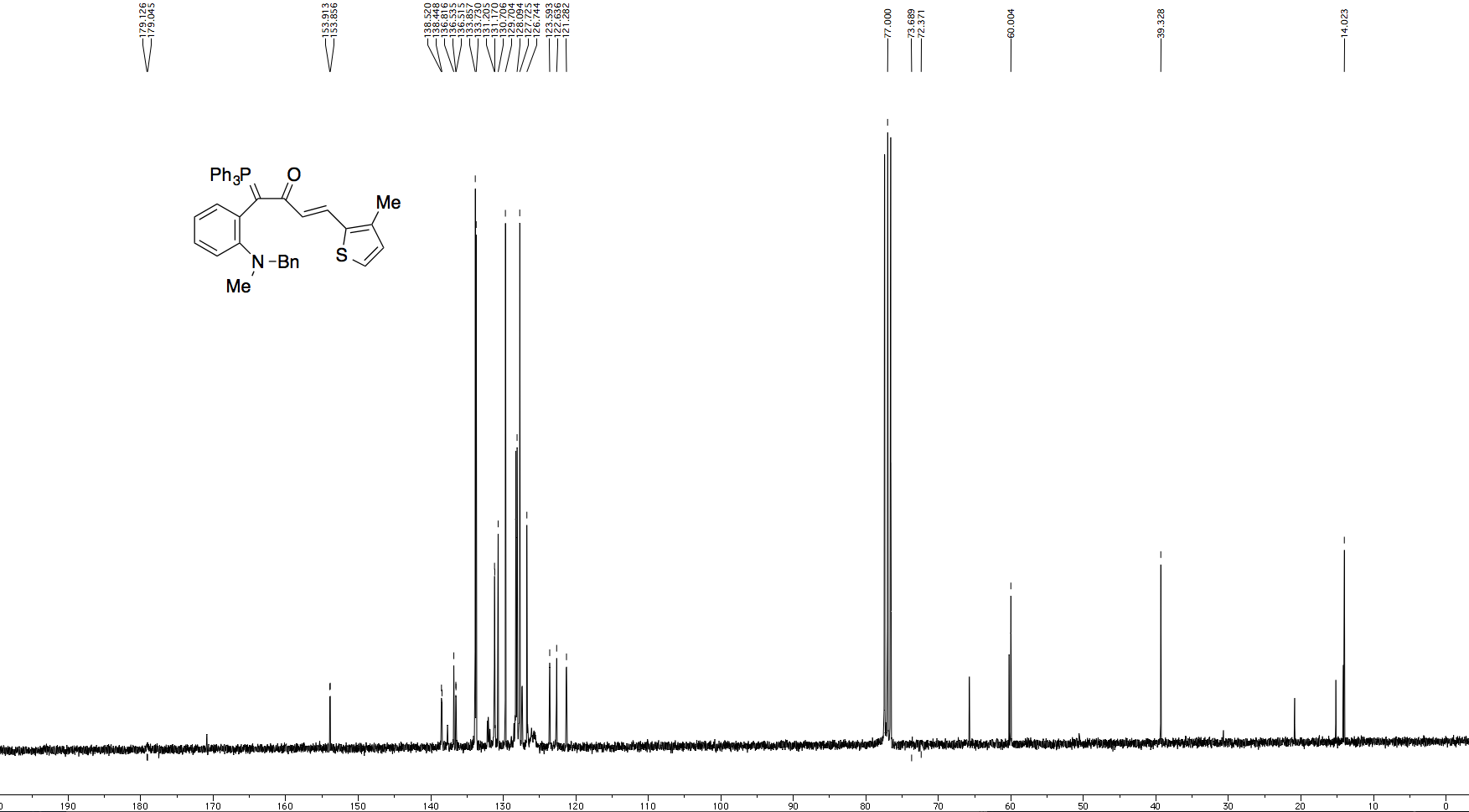


Figure S20. 400 MHz ^1^H NMR spectrum of **41**


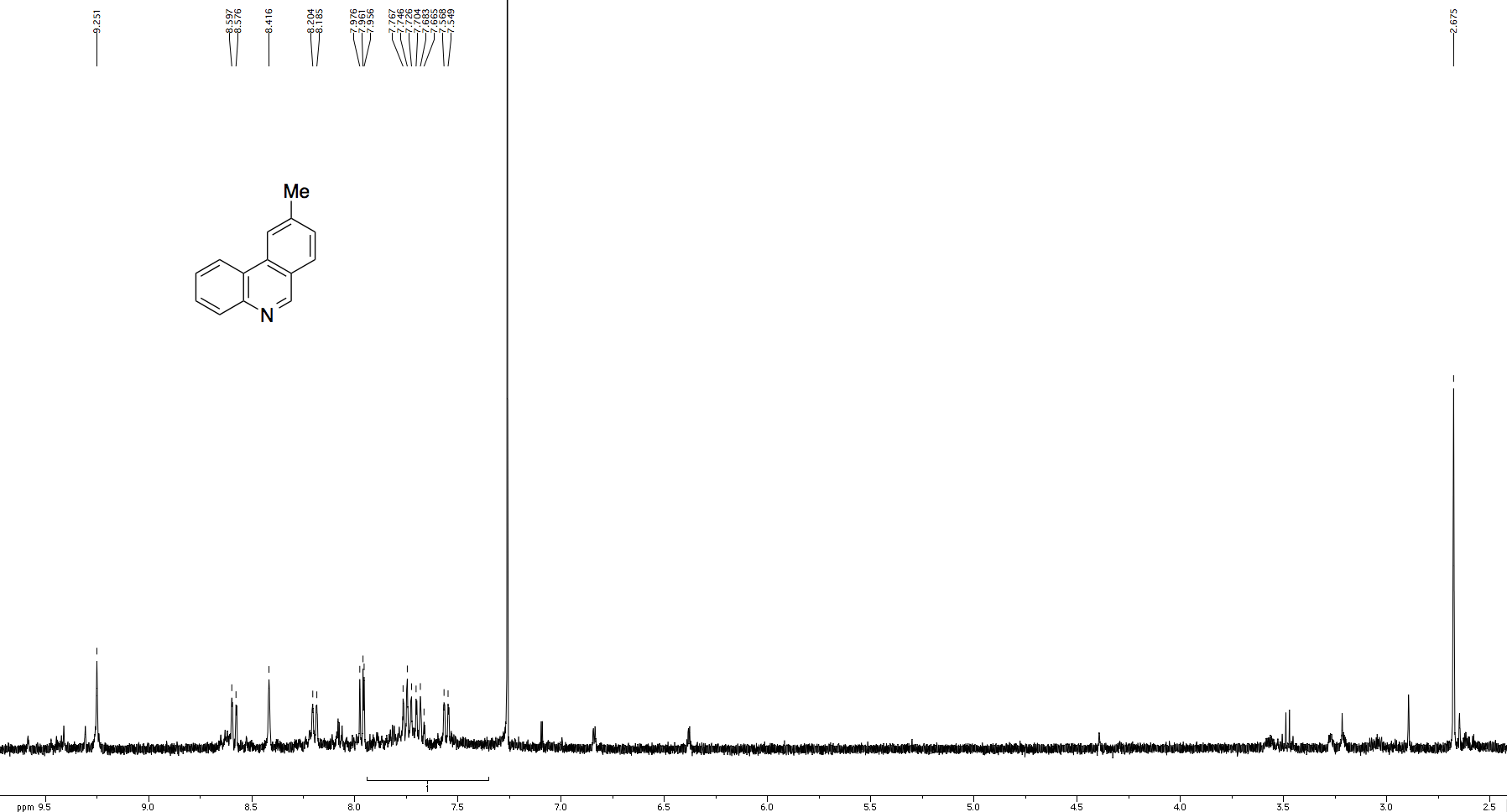


Figure S21. 400 MHz ^1^H NMR spectrum of **42**


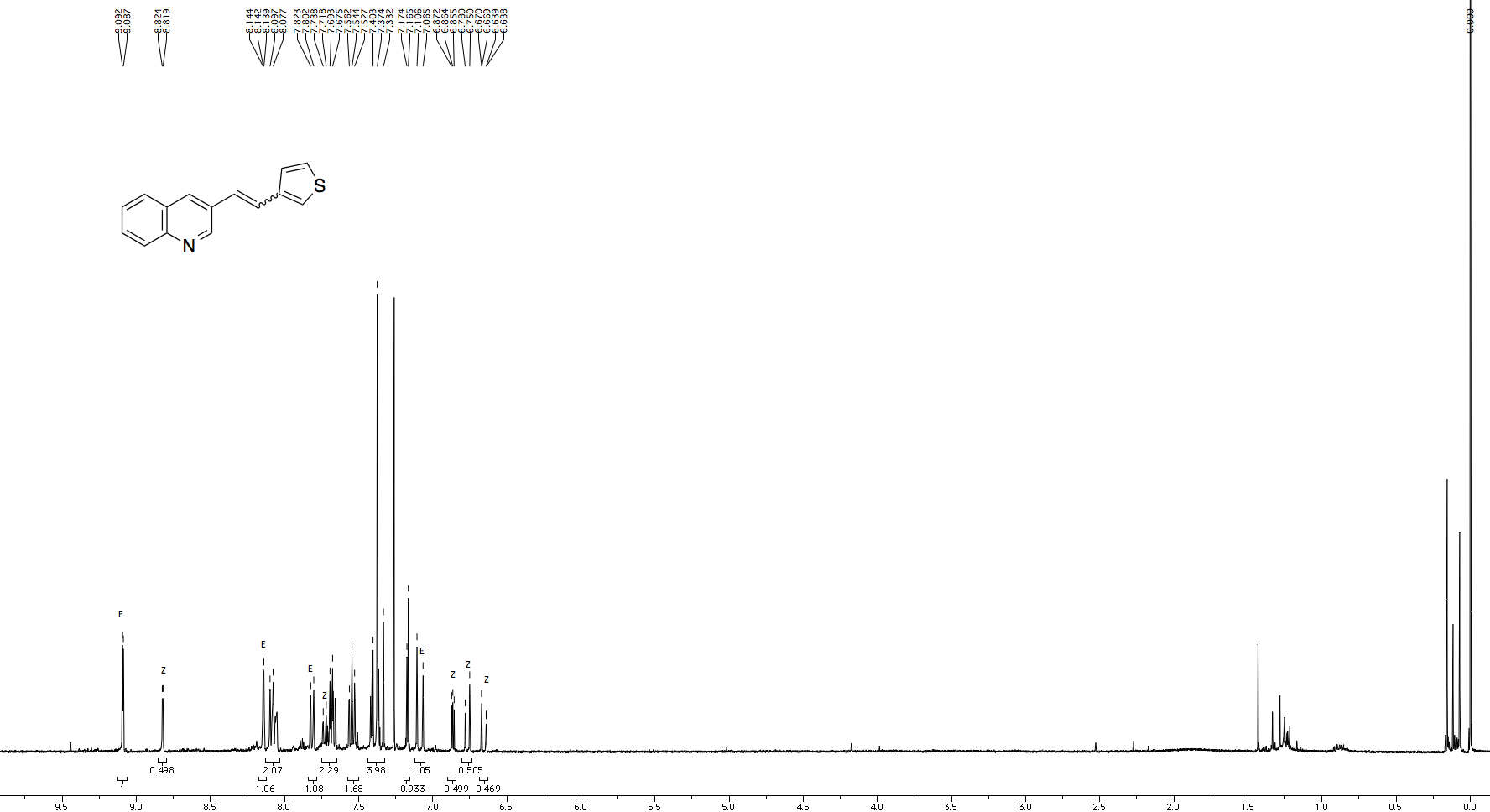


Figure S22. 400 MHz ^1^H NMR spectrum of **43**


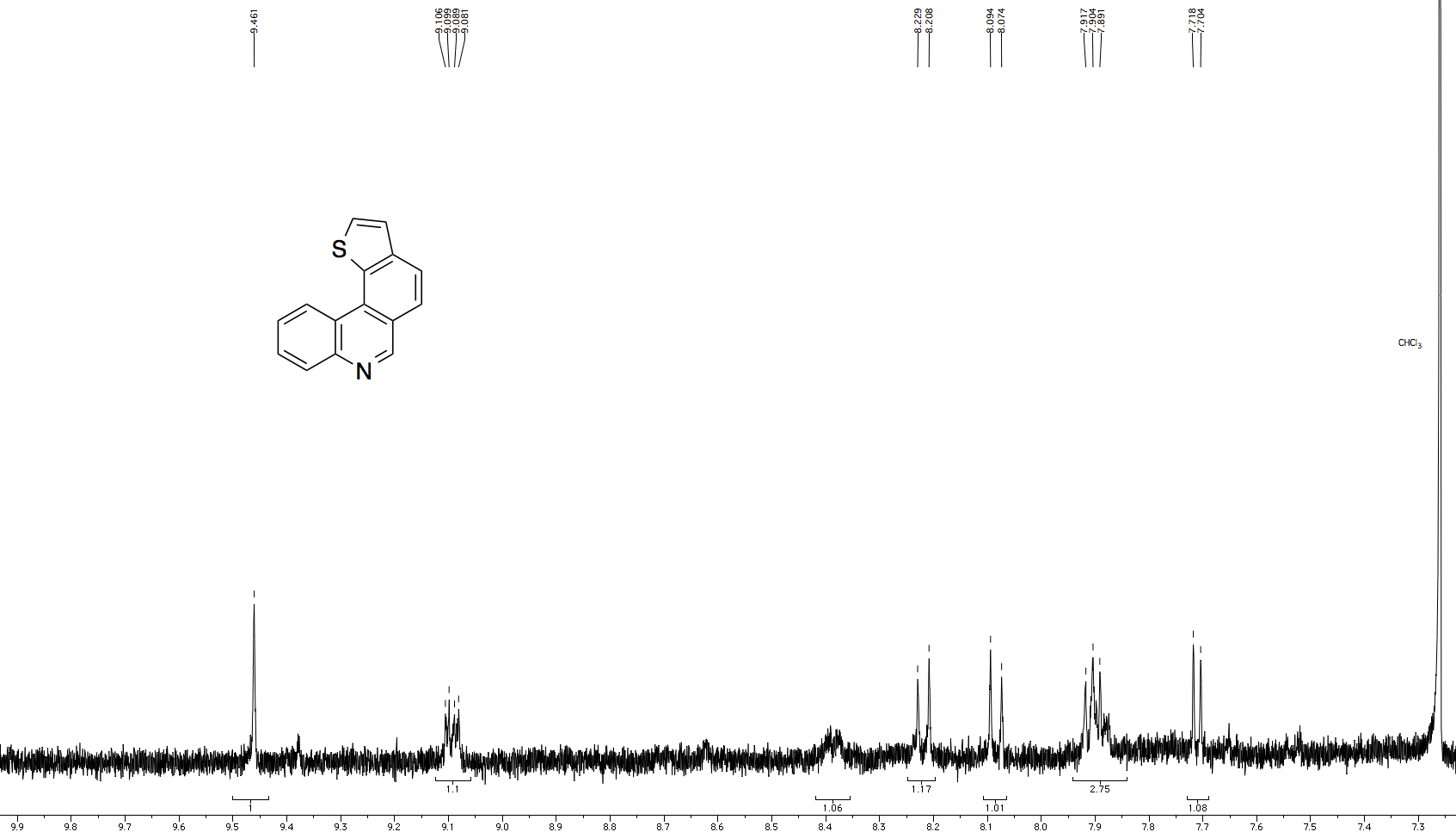


Figure S23. 400 MHz ^1^H NMR spectrum of **44**


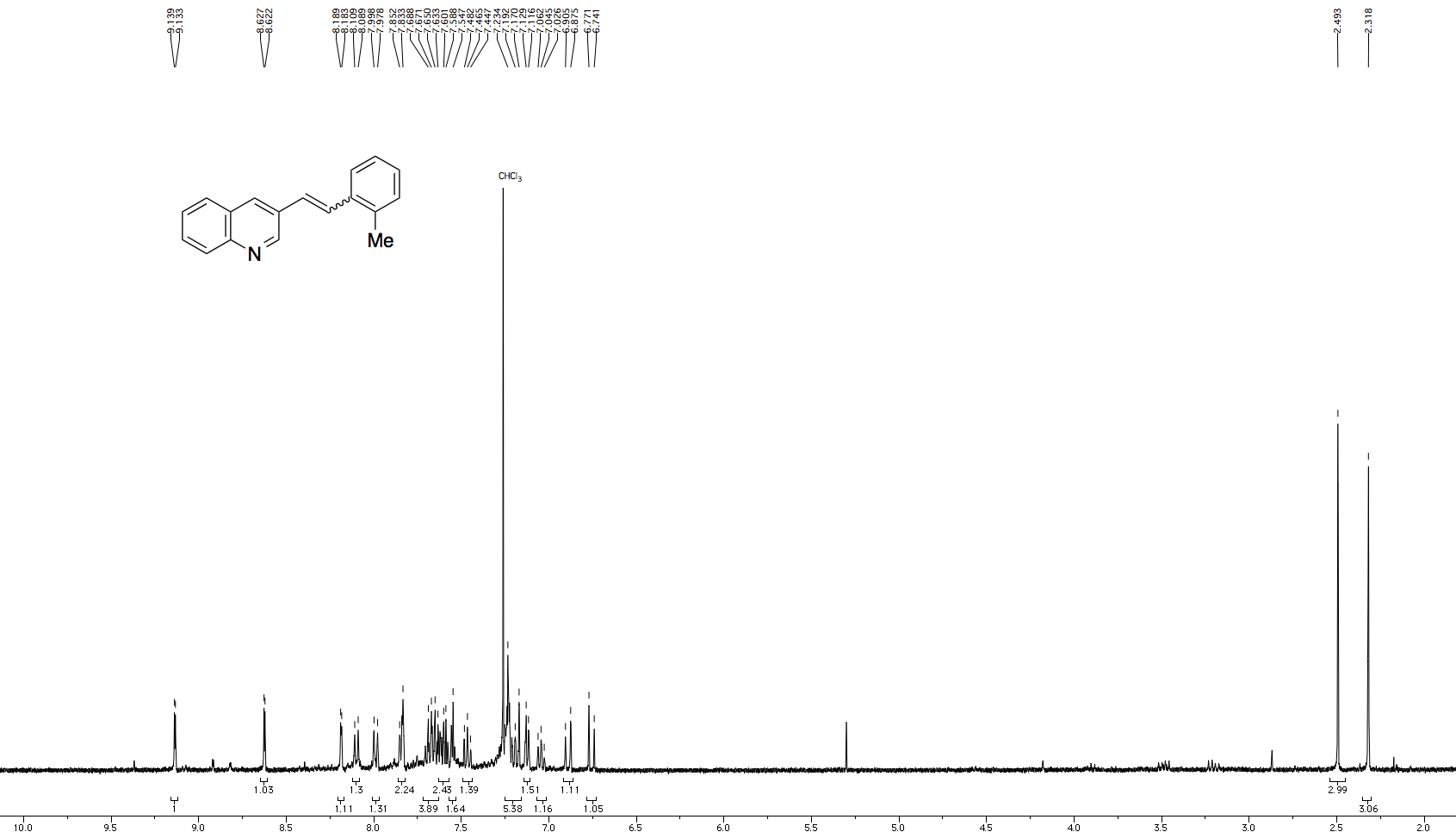


Figure S24. 400 MHz ^1^H NMR spectrum of **45**


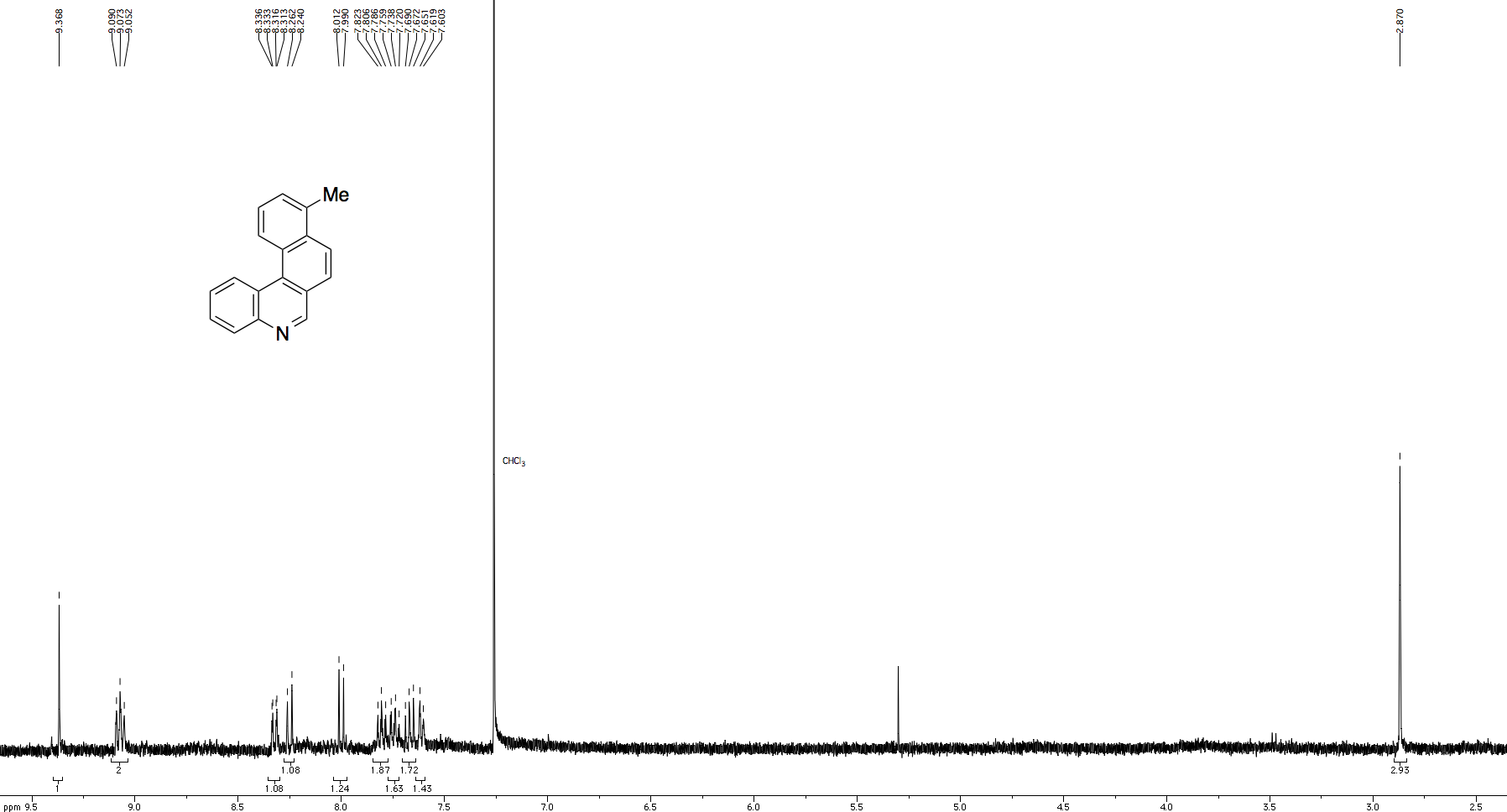


Figure S25. 400 MHz ^1^H NMR spectrum of **46**


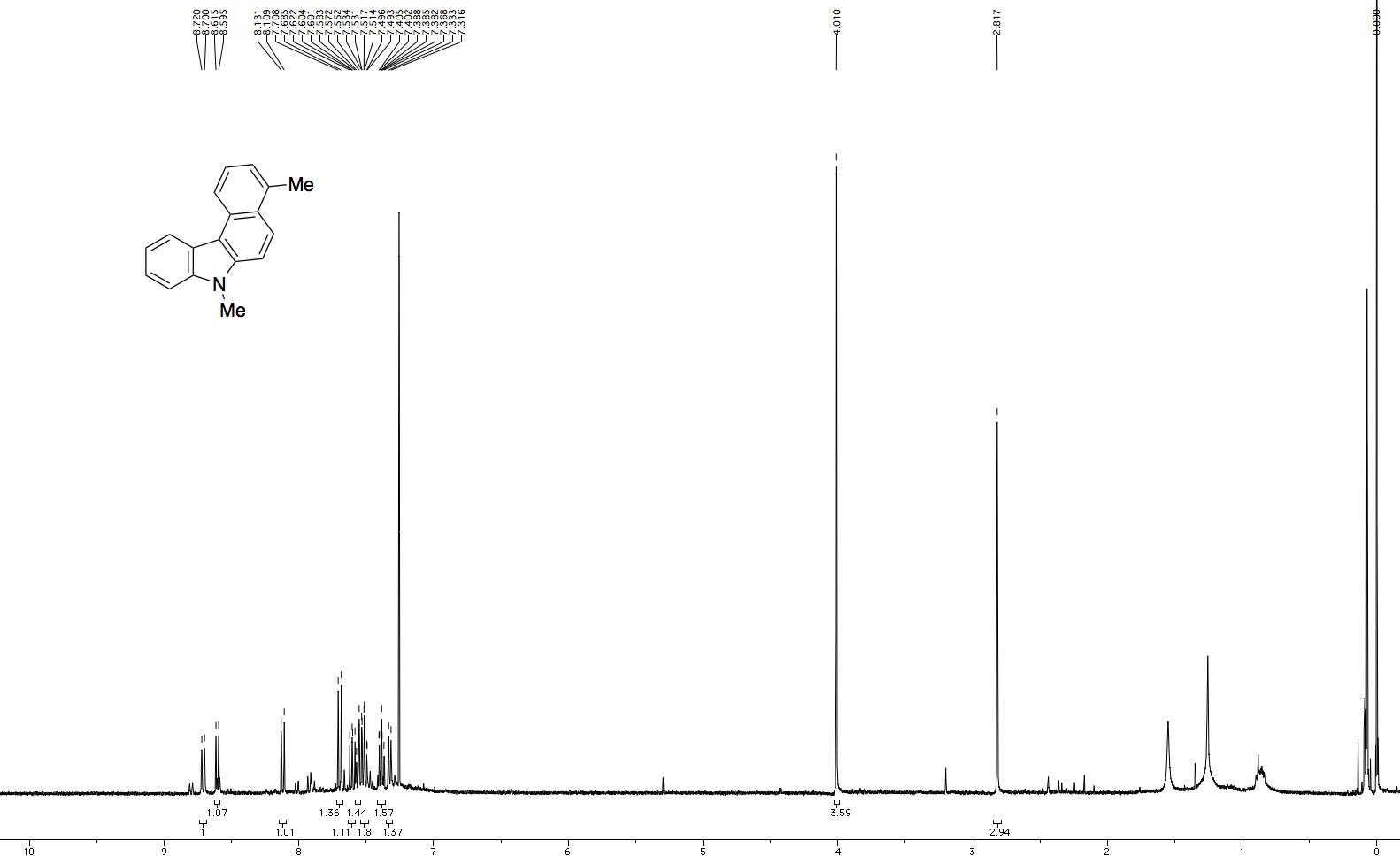


Figure S26. 75 MHz DEPTQ ^13^C NMR spectrum of **46**


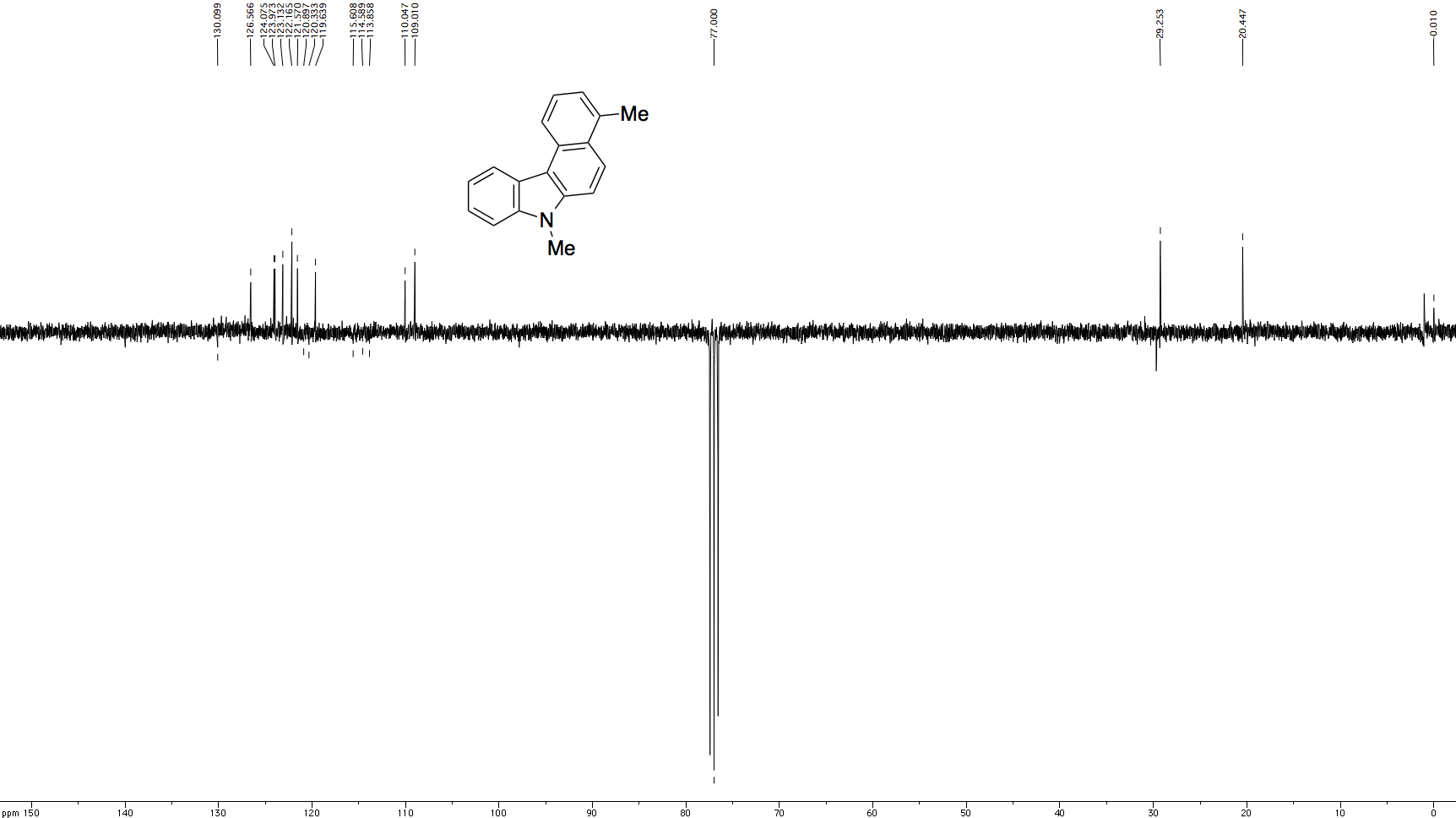


Figure S27. 400 MHz ^1^H NMR spectrum of **47**


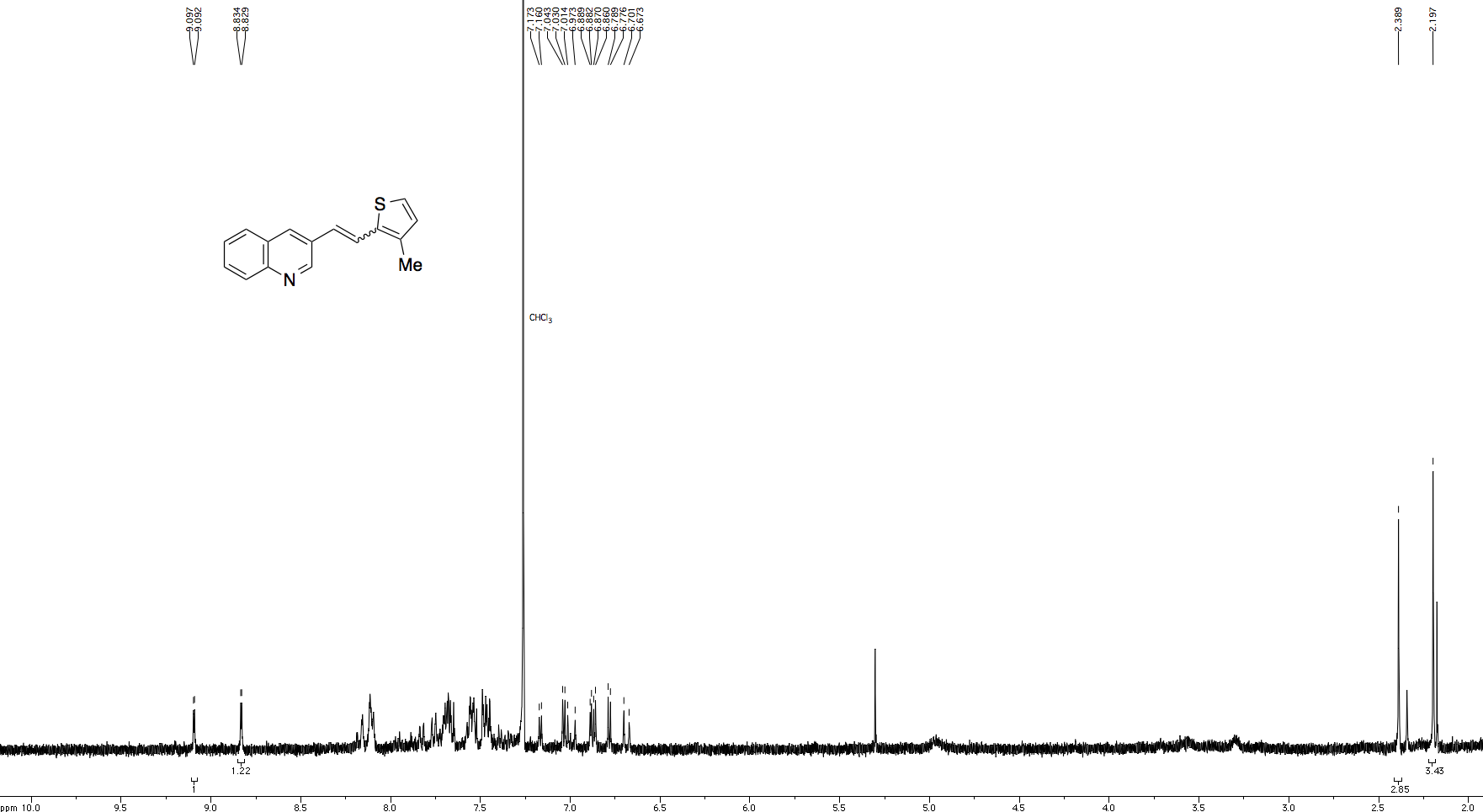

Supplement: Supplementary file 1 [file molecules-23-02153-s001.zip › supplementary-342311-revised/18Mol SupplMat/18Molecules_LM_RAA_suppl.docx]
